# Mechanistic Determinants of Oriented Enzyme Immobilization from Martini Simulations

**DOI:** 10.1021/acs.jpclett.5c03753

**Published:** 2026-02-09

**Authors:** Juan Carlos Jiménez-García, Nicoll Zeballos, Fernando López-Gallego, Xabier López, David De Sancho

**Affiliations:** † Polimero eta Material Aurreratuak: Fisika, Kimika eta Teknologia, Kimika Fakultatea, 226245 UPV/EHU & Donostia International Physics Center (DIPC), PK 1072, 20018 Donostia-San Sebastian, Euskadi, Spain; ‡ Center for Cooperative Research in Biomaterials (CIC biomaGUNE), Basque Research and Technology Alliance (BRTA), Paseo Miramon 194, 20014 San Sebastián, Spain; ¶ Ikerbasque, Basque Foundation for Science, 48013 Bilbao, Spain

## Abstract

Although enzyme immobilization is widely used in biotechnology, it still poses challenges as a result of the trade-offs among stability, activity, and surface interactions. Computer simulations offer a promising aid to exploring the effects of different immobilization sites and surface chemistry on both the conformational dynamics and catalytic activity of these biomolecules. Here, we introduce a protocol based on a structure-based version of the Martini coarse-grained simulation model (Go̅Martini) to explore how surface tethering geometry influences the structure and function of immobilized *Bacillus stearothermophilus* alcohol dehydrogenase (BsADH). We compare traditional His-tag tethering with two engineered histidine cluster variants, analyzing their behavior in both soluble and surface-tethered states. We find that cluster-based immobilization locally restricts flexibility in surface-contacting subunits while preserving the mobility of exposed regions, resulting in an enhanced conformational stability under thermal stress. Functional analyses reveal that the ethanol association rates remain largely unaffected by surface attachment, whereas the dissociation of NADH is significantly slowed, explaining the reduced catalytic efficiency. These trends align with experimental findings and highlight the predictive power of Go̅Martini simulations in capturing key functional trade-offs. Altogether, this work offers mechanistic insight into the rational design of immobilized biocatalysts and outlines a practical framework for in silico exploration of enzyme–surface systems.

## Graphical abstract

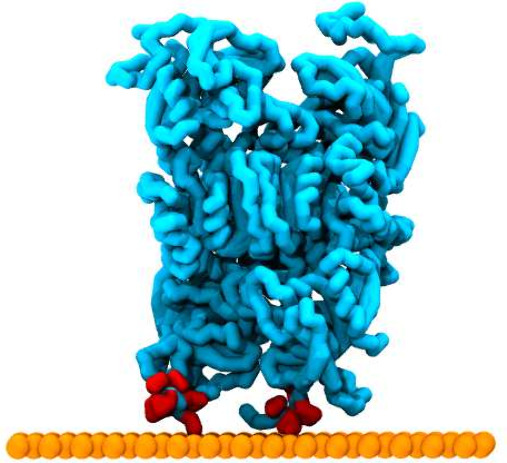

Enzymes are fundamental biological catalysts in biotechnology and green chemistry, performing highly specific reactions under mild and sustainable conditions. They are used in industrial sectors such as pharmaceuticals, food processing, fine chemical production, energy and sensing.^1–7^ However, their full potential remains underexploited, as many enzymes lose activity under harsh conditions, have short operational lifetimes, and are difficult to recover efficiently.^8–10^ Immobilization on solid supports is a proven strategy to overcome these limitations and enhance enzyme robustness.^11,12^ By tethering enzymes to insoluble matrices, separation from the medium is facilitated, structural stabilization improves, and reuse becomes feasible, lowering costs while increasing process efficiency.^13,14^ Furthermore, immobilization of enzymes enables the application of biocatalysis in flow as enzymes can be packed in reactor beads, attached to reactor walls, and grafted to reactor monoliths.^13,15–19^ However, immobilization can also induce conformational changes that reduce activity, particularly when the active site faces the support or when excessive rigidity limits catalysis.^14,20,21^ Traditionally, enzyme immobilization protocols have largely been empirical, and the design of materials and immobilization chemistries that stabilize enzymes while preserving their activity relies on a trial and error approach. As a result, enzymes are mainly immobilized through uncontrolled orientation, yielding unproductive enzymes with either unfavorable orientations or excessively rigid conformations. This leads to dramatic enzyme reductions.^12,22,23^ Therefore, achieving oriented immobilization has been pursued in advanced immobilization approaches to preserve both activity and stability in heterogenized biocatalysts.^24^

One of the most common ways to control enzyme orientation is through affinity peptide tags, typically the polyhistidine tag (His-tag), which binds metal ions such as Ni^2+^ or Co^2+^.^25,26^ His-tag immobilization allows simultaneous purification and attachment.^27–30^ However, the orientation through the His-tag is not always optimal to maximize the activity/stability balance as it can trigger an inactive conformation when bound to surfaces.^31^ This case is illustrated with enzymes where their N- or C-termini play a role in catalysis. To expand control, site-directed strategies have introduced alternative tethering residues at specific surface sites.^32–40^ Recently, a novel concept has emerged: engineering histidine clusters on enzyme surfaces to enable multidentate, orientation-controlled tethering.^39–41^ By placing several histidines in flexible regions distant from the catalytic site, enzymes can achieve stronger, better defined interactions with metal-chelating supports. Experimental studies show that such clusters improve retained activity and stability compared to terminal tags, likely due to more favorable orientation and reduced steric hindrance.^39–41^ These results highlight the potential of rational surface design to fine-tune the enzyme orientation, although the design principles remain poorly understood.

Despite extensive experimental success, the molecular mechanisms that govern enzyme-surface interactions are still elusive.^14,20,42^ Predicting how immobilization affects structure, flexibility, and catalysis requires atomistic insight that is difficult to obtain experimentally. Molecular dynamics (MD) simulations have therefore become essential tools for probing adsorption, orientation, and conformational effects at solid–liquid interfaces. All-atom MD studies have illuminated enzyme–surface interactions on materials such as silica, carbon, and self-assembled monolayers,^43–47^ but their computational cost limits systematic exploration across variants and time scales. Coarse-grained models (CG) address this limitation by reducing atomic detail while preserving key physicochemical features.^48^

Here, we study enzyme immobilization using Martini, a well-established CG model originally developed for lipids^49,50^ and later extended to proteins,^51^ which enables microsecond to millisecond simulations of large biomolecular systems. Due to the inability of the Martini model to fold proteins, several variants have been introduced, including elastic networks such as ELNEDIN,^52^ which preserves the 3D structure of the protein but restricts conformational transitions. Instead, here we employ the Go̅Martini model,^53^ which integrates Martini3 nonbonded interactions with a structure-based (i.e., Go̅-like) potential derived from the native contacts in the experimental structure of the protein.^54^ Unlike harmonic elastic networks, Go̅Martini stabilizes the native fold through Lennard-Jones interactions, allowing large-scale motions relevant to catalysis. Previous studies using Martini confirm the ability of the model to capture immobilization effects. Simulations of lysozyme adsorption revealed that hydrophobic surfaces promote unfolding and occlusion of the active-site, whereas hydrophilic surfaces preserve native orientation and activity.^55^ Other works demonstrated that properly tuned CG parameters reproduce reversible adsorption and pH-dependent desorption consistent with experiments.^56^ Together, these findings support the use of CG simulations to explore enzyme–surface mechanisms that govern orientation, stability, and catalytic accessibility.

In this work, we apply the Go̅Martini model to the tetrameric alcohol dehydrogenase from *Bacillus stearothermophilus* (BsADH), a robust biocatalyst of industrial relevance.^57–60^ ADHs catalyze reversible oxidation–reduction between alcohols (A_red_) and aldehyde/ketones (B_ox_)­

1 $$A_{\mathrm{red}}+{\mathrm{NAD}}^{+}⇌B_{\mathrm{ox}}+\mathrm{NADH}$$

and rely on NAD^+^/NADH as cofactors. In Figure 1A we show a representation of the crystal structure of BsADH,^61^ which we modeled in soluble and immobilized forms following Zeballos et al.^40^ In the experiments, different variants of BsADH containing either an N-terminus His-tag or His-cluster at different enzyme regions were immobilized on porous agarose beads functionalized with metal chelates through affinity guided immobilization based on metal-imidazol coordination bonds between the enzyme and the support surfaces. In Figure 1B we show a representation of the enzyme using the Go̅Martini beads. In this work, native contacts were identified using the OV+rCSU scheme (see Figure 1C,D), which combines geometric and chemical criteria for physical realism.^53^ Following the experimental design of Zeballos et al.,^40^ we examined three enzyme variants: (i) the wild-type bearing an *N*-terminal His-tag (Htag) and (ii–iii) two engineered mutants containing surface His clusters (H3: Q8H/K10H/E11H and H4: E8H/E11H/E265H/E266H, see Figure 1E). By comparing His-tag and His-cluster tethering, we investigate how immobilization geometry modulates enzyme structure, flexibility, and cofactor accessibility, providing molecular insight into the experimentally observed activity hierarchy.

**Figure 1:**
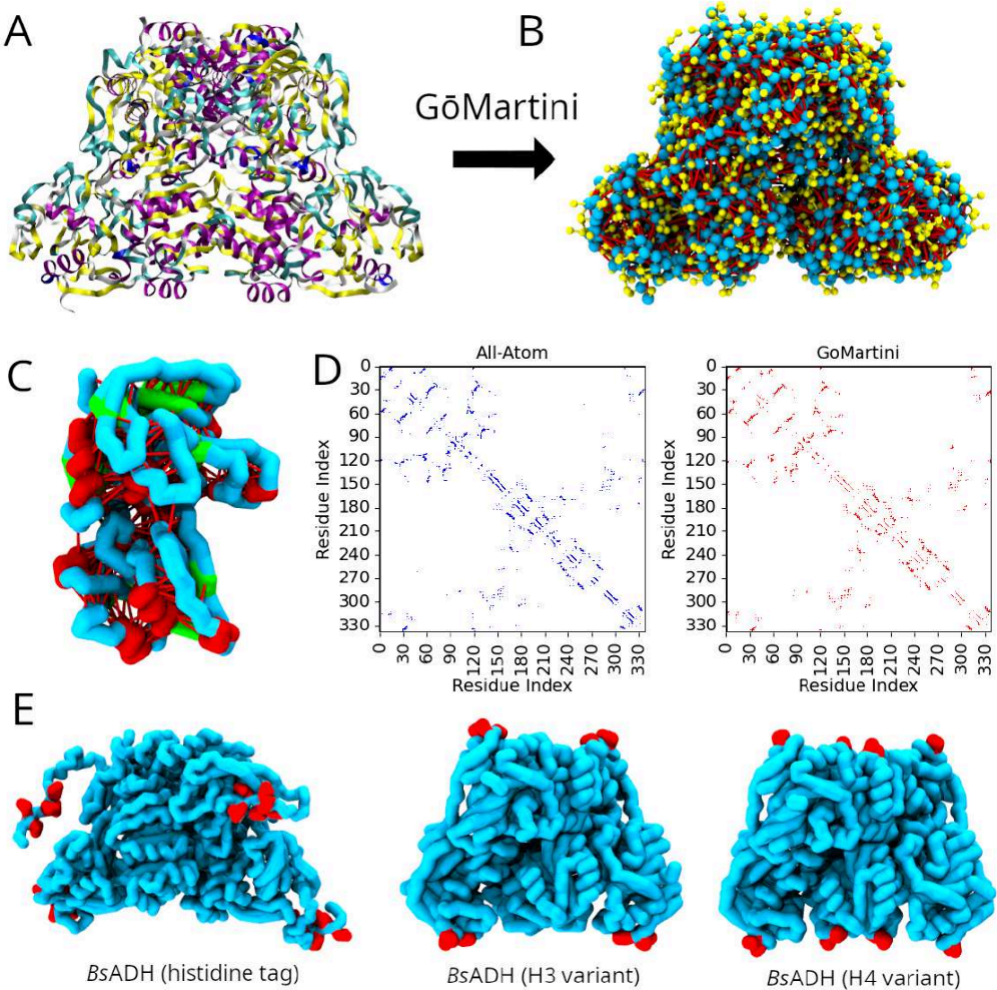
(A) All-atom structure of tetrameric BsADH (PDB: 1RJW), with secondary structure elements highlighted: α-helices represented in purple and β-strands shown as yellow ribbons. (B) CG Go̅Martini representation showing backbone (blue) and side-chain (yellow) beads, with native contacts in red. (C) Single-chain CG model highlighting α-helices (red) and β-sheets (green). (D) Comparison of native contact maps for chain A in the all-atom (left) and CG (right) representations. (E) BsADH variants: His-tagged WT (left) and engineered histidine-cluster mutants H3 (center) and H4 (right). Histidine beads are shown in red.

For immobilized systems, we developed a protocol to model surface interactions and tethering. The immobilization surface was modeled as a hydrophilic agarose-like matrix composed of fixed Martini P4-type beads arranged in a hexagonal lattice with a spacing of 0.47 nm. P4 beads accurately represent highly polar, hydroxyl-rich moieties characteristic of polysaccharides, such as agarose. While the flat surface utilized in this study provides a controlled environment to isolate the effects of tethering geometry, we acknowledge it represents a simplified model of the heterogeneous agarose matrix. This representation prioritizes the dominant physicochemical interactions between the enzyme and the hydrophilic saccharide units of the support, although it does not account for the inherent surface roughness or polymer chain mobility of the physical beads. Future iterations of this model could incorporate more complex surface architectures combined with saccharide-specific Martini parameters^62^ to further refine the description of the enzyme–support interface.

The two-phase immobilization process is summarized in Figure 2. First, we used a pulling force to induce a rapid approach of the enzyme to the surface (blue), and then we switched on a harmonic tethering potential acting on the histidine residues (red). Immobilized systems were simulated for a total of 1.2 μs, comprising a 200 ns deposition phase allowing lateral diffusion, followed by a 1 μs tethered phase under residue restraints. As the protein approaches and is tethered to the surface, we observe a transient increase in the RMSD, indicating a conformational perturbation as the protein engages with the surface. After this initial interaction, the RMSD stabilizes, reflecting structural adaptation and equilibrium under tethered conditions. Figure 2B illustrates representative snapshots at selected time points (0, 50, 200, and 400 ns), visually capturing the gradual deposition and tethering of the His-tagged enzyme to the support. Additional details about the implementation of the simulation model can be found in the .

**Figure 2:**
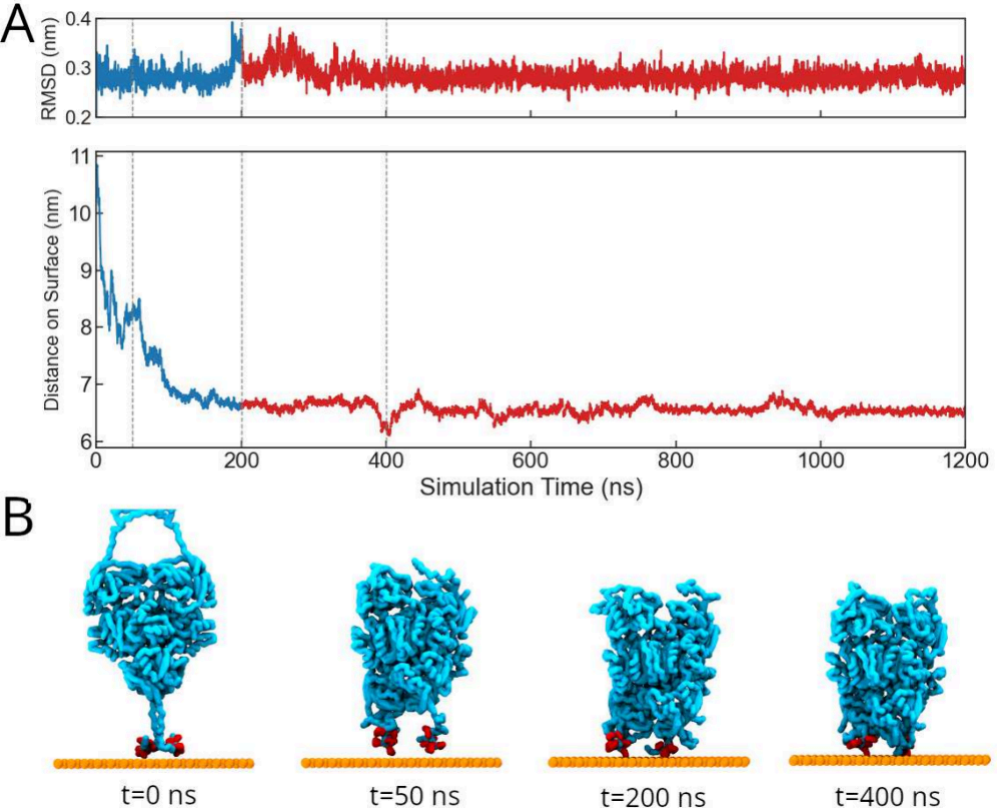
Immobilization modeling and surface interaction protocol. (A) Temporal evolution of the enzyme–surface distance (bottom) and backbone RMSD (top) showing adsorption and equilibration phases. (B) Representative snapshots illustrating the gradual deposition and tethering of the His-tagged enzyme onto the hydrophilic agarose-like surface.

We first ensured that unspecific adsorption of the enzyme onto the agarose surface does not occur under our modeling conditions. Control simulations without tethering restraints showed no stable interaction between the protein and the support (see ), confirming the need for explicit immobilization strategies to achieve surface tethering in silico. In the experimental system, by contrast, immobilization is mediated by metal–histidine coordination between engineered His residues and Co^2+^-activated agarose. The behavior observed in the simulations is consistent with experimental observations, where BsADH does not immobilize on agarose supports in the absence of the His-tag or His-cluster that allow the metal–histidine coordination, despite the hydrophilic nature of the surface. The lack of spontaneous adsorption in our model therefore reflects a physically meaningful outcome rather than a limitation, ensuring that immobilization arises exclusively from the intended chemical tethering mechanism. To assess how explicit tethering affects structural dynamics, we first analyzed each BsADH variant in solution, in the absence of both surface and restraints, to establish a baseline for comparison (see ). We first computed the root-mean-square deviation (RMSD) of the backbone beads for all BsADH variants at 300 K. All systems displayed stable behavior in the simulations of 1 μs, with very similar average RMSD values around ∼2 Å (); therefore, the modifications we have introduced do not affect the enzyme fold (see also the values of the RMSF for each of the chains in all variants in ) in agreement with the experimental kinects determined for these variants.^40^

Next, we analyzed the structural dynamics of surface-immobilized BsADH variants. The *RMSD* profiles (Figure 3A) indicate that histidine cluster variants (H3 and H4) exhibit lower structural deviations than the His-tagged enzyme. Despite all immobilized constructs adopting asymmetric orientations, their contact patterns differ: in H3 and H4, subunits A and D are tethered to the surface, whereas in the His-tagged system, the tethered subunits are A and C (Figure 3C–E). The remaining chains are fully solvent-exposed. This differential tethering leads to subunit-specific effects. Subunits in contact with the surface show reduced *RMSD* and *RMSF* values. On the other hand, exposed subunits maintain fluctuation levels comparable to those of the soluble enzyme, indicating that immobilization effects are localized and do not propagate through the tetramer. The *RMSF* profiles in Figure 3B further reveal that the H3 and H4 variants exhibit reduced flexibility in regions containing engineered histidines (8H/10H/11H in H3 and 8H/11H/26H/26H in H4), as well as the C-termini region, specifically in surface-tethered subunits, in agreement with more rigid site-specific tethering. In contrast, the His-tagged enzyme displays a mobility comparable to that of the untethered form, suggesting weaker conformational restriction.

**Figure 3:**
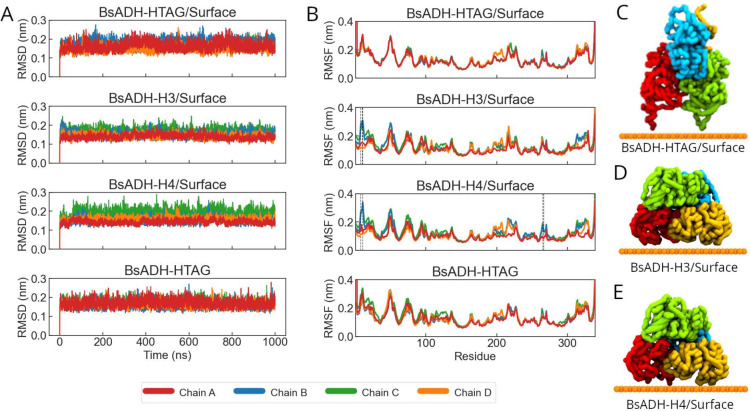
(A) Backbone *RMSD* and (B) *RMSF* profiles of individual chains for immobilized BsADH variants over 1 μs. Dashed black lines indicate the residue used for immobilization in each variant. (C–E) Final conformations of the immobilized BsADH-Htag (C), BsADH-H3 (D), and BsADH-H4 (E) systems after 1 μs of simulation. Protein color scheme: chain A in red, chain B in cyan, chain C in green, and chain D in yellow. Orange beads represent the immobilization surface.

While chain A is tethered in all constructs, only in H3 and H4 does it exhibit a marked reduction in flexibility, highlighting the stabilizing effect of multivalent cluster tethering. Conversely, chain B, which remains distant from the surface in all systems, shows nearly identical *RMSF* profiles across variants, reinforcing the localized nature of the stabilization. Together, these results demonstrate that histidine-based immobilization induces nonuniform stabilization across the ADH tetramer. Engineered clusters provide conformational restriction more effectively and localized than flexible His-tags, which may impact enzymatic stability and active-site accessibility, as discussed in the following paragraphs.

To assess conformational stability under thermal stress, we performed MD simulations reducing native contact energy using a lower scaling factor (λ = 1.2 instead of the standard λ = 1.5) to allow partial unfolding at lower temperatures. At room temperature (300 K), all systems remained structurally stable, reproducing the behavior observed under the standard conditions (see ). The effect of immobilization was evident in the restricted motion of subunits tethered to the surface (see ). In Figure 4 we compare the average thermal response from multiple runs of both soluble and immobilized BsADH variants at *T* = 500 K. In solution, BsADH-Htag exhibits the fastest departure from the native state, while H3 and H4 are more resistant to thermal stress. Upon immobilization on a hydrophilic surface, all variants showed enhanced stability with H3 and H4 again showing the greatest resistance to unfolding. Representative conformations shown in Figure 4B,C illustrate that soluble enzymes undergo partial domain separation, whereas immobilized constructs—particularly those tethered through histidine clusters—preserve a more native-like quaternary arrangement.

**Figure 4:**
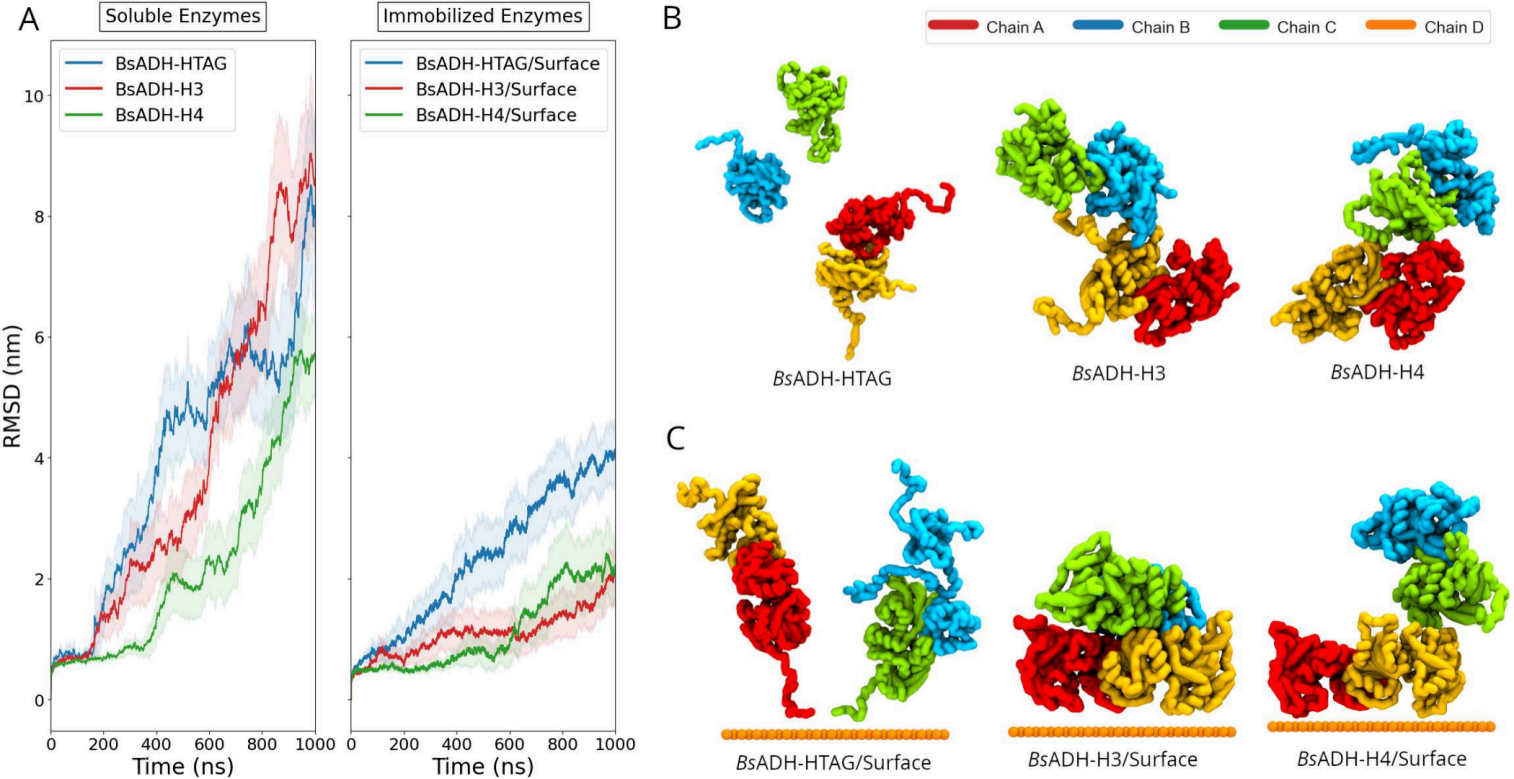
(A) Backbone *RMSD* of soluble and immobilized (BsADH-Htag/Surface, BsADH-H3/Surface, and BsADH-H4/Surface) variants at 500 K. Representative conformational changes from the soluble simulations (B) and immobilized systems (C).

To quantify thermal melting, we have estimated free-energy landscapes *F*(*Q*) as a function of the fraction of native contacts *Q* at 300, 400, and 500 K (see ). We note that despite the weakening of the interaction energies with λ = 1.2, complete unfolding is prevented by the intrinsic constraints of the Go̅Martini model. Recent refinements in the contact-map definitions of Go̅Martini^63^ may also be useful for future work. Nevertheless, the current model allows us to capture the relative differences between the variants. At 300 K, all systems exhibited a well-defined minimum at *Q* ≃ 0.98, indicating stable folded conformations. At 400 K, only a minor decrease in *Q* was observed, confirming that the integrity of the protein remained largely preserved. However, at 500 K, the soluble enzymes showed broader energy minima and reduced *Q* values, consistent with the dissociation of the subunits, while the immobilized variants retained higher *Q* values and deeper minima, denoting greater structural preservation.

Among immobilized systems, H3 exhibited a slightly higher thermal resistance than H4, suggesting a more favorable tethering geometry that stabilizes the tetrameric assembly. These results are consistent with the behavior reported by Zeballos et al.^40^ and the experimentally determined melting temperatures (*T*_m_) of the immobilized enzymes determined through differential scanning fluorimetry (DSF) (see ). H3 and H4 display *T*_m_ values up to 17 and 10 °C higher than the *T*_m_ value displayed by the His-tag enzyme immobilized on the same support. The increase in *T*_m_ has also been experimentally observed in other enzyme classes immobilized on solid surfaces. The stabilization degree depends on both the enzyme orientation and the physicochemical properties of the surface where the enzyme is immobilized.^64,65^ Multivalent interactions between enzyme and support surface are often responsible for the increase of the thermodynamic stability due to structural rigidification. Here, the multivalent attachment between the His-clusters through very short spacer arms between enzyme and support surfaces supports the higher thermal stabilization observed with the variants H3 and H4 compared to the His-tagged enzyme.

Next, we focus on the functional impact of enzyme immobilization. Although recent work has incorporated reactivity into Martini,^66^ the resolution of the model is not yet sufficient to study full enzymatic reactions. Therefore, we assume that the intrinsic catalytic transformation is not affected by the immobilization. Instead, we focus on the two stages that Martini can reliably describe and that are expected to be most sensitive to immobilization: substrate entry and cofactor/product release (see Figure 5A). Specifically, to analyze the substrate entry, we run simulations of the NAD^+^-bound enzyme in the presence of 10 ethanol molecules. The cofactor was represented using the Martini parameters reported by Barriga-Alves et al.,^67^ and ethanol was modeled as a single bead^68^ (see further details in the ). Ethanol binding events were determined using distance-based contact analysis. Binary contact signals were constructed per frame in both soluble and immobilized systems (see ). Ethanol–cofactor distances were converted to binary contact traces and smoothed to suppress transient collision spikes, retaining only persistent binding events. All systems exhibited transient ethanol interactions; however, their frequency, persistence, and stability varied markedly depending on the immobilization strategy.

**Figure 5:**
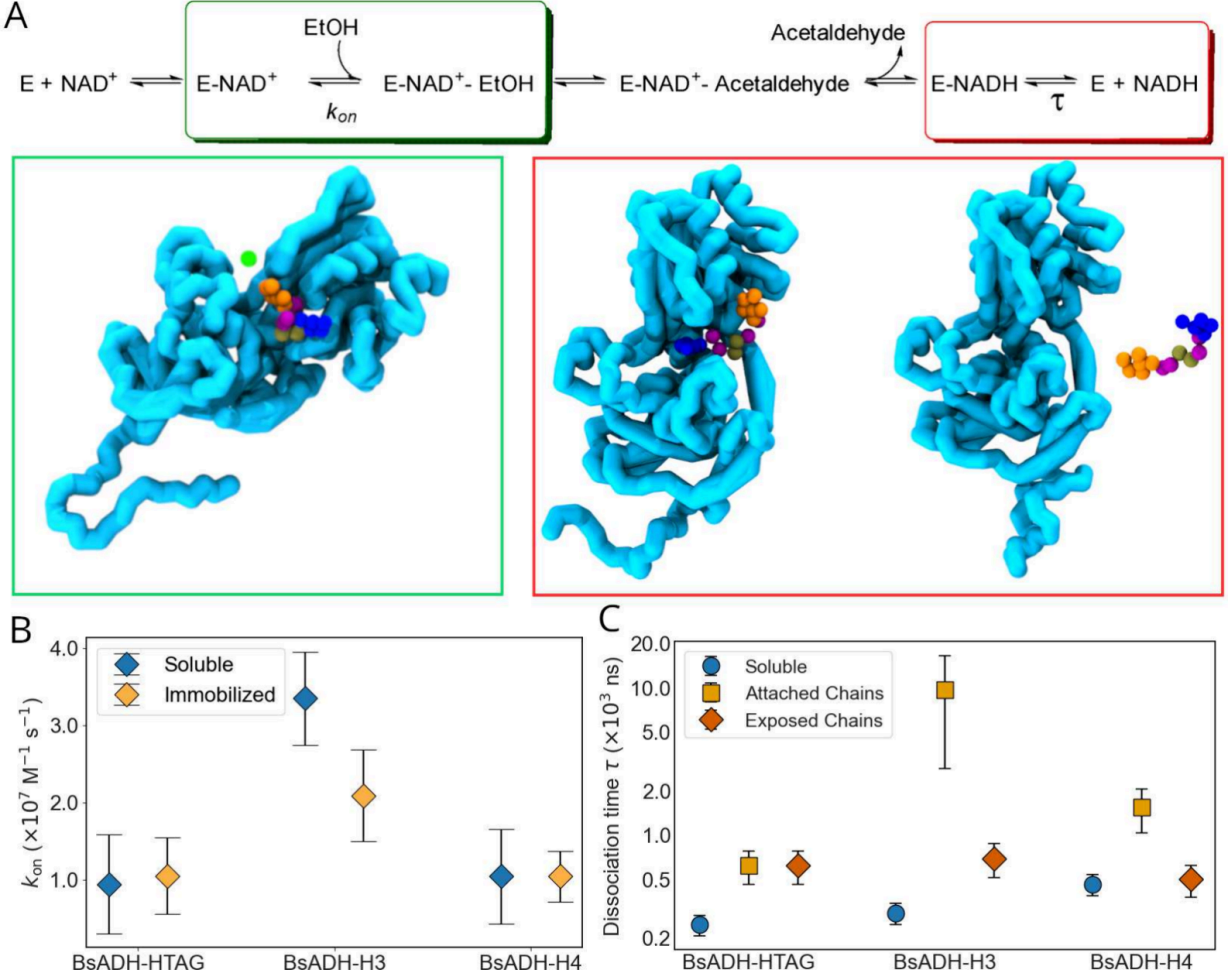
(A) Catalytic scheme illustrating the ordered sequence of substrate binding and product release steps in the BsADH mechanism, with representative conformations of the enzyme (cyan) corresponding to the ethanol association (*E* – NAD^+^ · EtOH, green box), NADH-bound (*E* – NADH, red box), and NADH-unbound (*E* + NADH, red box) states. Cofactors and substrates are shown as colored beads. (B) Ethanol association rate constants (*k*_on_) for soluble and surface-immobilized BsADH-HTAG, BsADH-H3, and BsADH-H4 variants, obtained from contact-based detection of binding events. (C) NADH dissociation times (τ) computed for soluble enzymes and for surface-immobilized chains classified as blocked or solvent-exposed.

From the number of identified binding events, the ethanol association rate constant (*k*_on_) was estimated from each of the simulation runs as *k*_on_ = *n*_bind_/(*t*_sim_ · [EtOH]), where *n*_bind_ is the number of binding events, *t*_sim_ is the simulation time, and we use the concentration of ethanol in the simulation box ([EtOH] = 6.3 mM for BsADH-HTAG and 9.6 mM for BsADH-H3 and BsADH-H4). In Figure 5B, we compare the substrate association rate constants (*k*_on_) for the soluble and surface-immobilized BsADH variants. The His-tagged enzyme consistently exhibits the lowest *k*_on_ values in both states, suggesting that the flexible His-tag tail transiently occludes the entrance to the catalytic pocket and thereby limits productive binding encounters. Among all systems, the engineered BsADH-H3 variant displays the highest *k*_on_ values in solution, consistent with a more accessible active-site environment. Upon immobilization, however, BsADH-H3 shows a marked decrease in *k*_on_, implying that surface attachment restricts substrate diffusion and orientation near the catalytic site. In contrast, the immobilized H4 and HTAG variants retain *k*_on_ values comparable to those of their soluble counterparts, suggesting that the influence of the surface is less pronounced or not effectively captured under the simulated conditions. Nevertheless, BsADH-H3 maintains *k*_on_ values higher than those of both H4 and HTAG in the immobilized state. These results are consistent with the 3 times higher apparent *k*_cat_ of H3 variants in the oxidation of ethanol compared to the His-tagged enzymes, both immobilized on the same support (agarose-based microbeads functionalized with cobalt chelates; see in Zeballos et al.^40^), reflecting a qualitative agreement between microscopic substrate entry and overall catalytic efficiency.

To further investigate the functional consequences of enzyme immobilization, we also study the dissociation of NADH once the ternary complex is disassembled and the acetaldehyde is released. This step may become the rate-limiting step in enzyme catalysis. This has been observed for other immobilized enzymes where the enzyme interaction with the solid surface slowed the product release from the active site, decreasing the catalytic constant of the immobilized biocatalys.^67,69^ In this case simulations were run starting with NADH located in the active site, and characteristic dissociation times (τ_ML_) were estimated using a maximum-likelihood estimator^70^ that considers both completed dissociation events and trajectories in which dissociation was not observed (see and Supporting Methods for additional details on the calculations). As shown in Figure 5C, all soluble variants have comparably short cofactor residence times. This behavior suggests that despite the modifications the enzymes preserve a dynamic and accessible active-site in solution, which promotes rapid cofactor release and exchange, essential steps for sustaining catalytic efficiency under turnover conditions. These results align well with experimental findings,^40^ which show that the histidine clusters have minimal impact on the enzymatic activity of ADH. In solution, both variants exhibit comparable *k*_cat_/*K*_M_ values for ethanol oxidation using NAD^+^ as cofactor. In contrast, the immobilized variants displayed markedly prolonged NADH dissociation times, consistent with their reduced catalytic activity. Experimental measurements reported apparent *k*_cat_/*K*_M_ values that are 1–2 orders of magnitude lower for immobilized enzymes compared to their soluble counterparts. This suggests that surface tethering may impose spatial restrictions or conformational constraints on the enzyme, thereby hindering NADH release and ultimately limiting turnover rates. Among the immobilized systems, BsADH-Htag and BsADH-H4 showed moderate increases in cofactor retention, whereas BsADH-H3 displayed a significantly more pronounced delay in NADH dissociation. To determine whether these effects were simply caused by the physical blocking of the active site, we carried the same analysis only on the exposed chains of the ADH tetramer in the immobilized systems. Even these accessible chains showed slightly longer dissociation times compared to those of their soluble counterparts (see Figure 5C). These results suggest that, in addition to steric effects, immobilization may influence the enzyme’s conformational dynamics that impact in the microkinetic parameters.

In summary, this study investigated how different orientations of the same enzyme immobilized through the same chemistry influence the structure and function of BsADH. Using Go̅Martini coarse-grained simulations, we compared conventional His-tag tethering with two rationally designed histidine clusters (H3 and H4) in soluble and immobilized forms. We find that immobilization modulates the conformational landscape of BsADH. Cluster-based anchoring (H3, H4) enhanced structural stability by restricting the flexibility of surface-contacting subunits while maintaining mobility in solvent-exposed regions, while His-tag tethering led to higher structural heterogeneity. Under thermal stress, H3 and H4 retained a greater fraction of native contacts, in agreement with experimental thermostability trends. Nevertheless, the Go̅Martini model is inherently native-centric and may restrict conformational sampling in multichain proteins, leading to a possible overestimation of absolute stability arising from the high number of stabilizing native contacts. For this reason, the stability trends reported here are intended to be interpreted comparatively across variants rather than as absolute measures. Future developments in native-centric Martini-based models, including refined contact-map definitions,^63,71^ may further improve the description of thermal unfolding processes.

From a functional perspective, ethanol-binding analyses reveal that immobilization modulates substrate association kinetics differently across variants. BsADH-H3 experiences the strongest immobilization-dependent reduction in *k*_on_, whereas H4 and HTAG display *k*_on_ values closer to those of their soluble forms, suggesting that orientation effects are more pronounced in H3. Exploring bulkier alcohols, such as benzyl alcohol, may further clarify how steric factors interact with immobilization geometry to shape binding kinetics and catalytic efficiency. In contrast, NADH dissociation is consistently slowed by immobilization across all variants, in agreement with experimentally observed reductions in catalytic turnover.

Our calculations with the Martini model show that the H3 variant gains stability upon immobilization, but this increased rigidity compromises NADH release during the final step of the catalytic cycle. When saturated with an oxidized cofactor, however, this orientation facilitates alcohol binding. Region-directed immobilization through the H8/H10/H11 cluster in H3 likely introduces steric restrictions that hinder NADH departure once the cofactor is release. These effects align with the widely reported activity–stability trade-off in immobilized enzymes,^72^ which may be partially mitigated by entropy gains associated with local heat uptake during catalysis.^73^

While the present work employs a flat agarose-like surface as a controlled reference model, extending this framework to more complex and heterogeneous surface architectures will be valuable for future studies addressing adsorption-driven or confinement-assisted immobilization mechanisms. In addition, our model assumes that cobalt chelates not involved in enzyme anchoring negligibly engage in nonspecific interactions with other residues exposed on the enzyme surface. This simplification was implemented because agarobiose repeating units are present at densities 3 orders of magnitude higher than cobalt chelates, based on the composition of 6% agarose microporous beads used in this study (cobalt-chelate density = 15 μmol·g^–1^). Consequently, we assume that the dominant nonspecific interactions between the surface and the enzyme are governed by the agarose-like surface, simulated as P4, rather than metal chelates. This assumption is consistent with observations reported in seminal studies on His-tag purification, where only His-tagged proteins bind surfaces functionalized with metal chelates.^74,75^

Together, these results highlight a key design principle: rational, site-specific tethering through engineered histidine clusters provides a more favorable balance between structural stability and catalytic accessibility in comparison to conventional His-tag immobilization. These computational trends are consistent with new and previously reported experimental data from Zeballos et al.,^40^ supporting Go̅Martini as a powerful framework to dissect mechanistic determinants of enzyme immobilization and potentially guide the rational optimization of immobilization geometry, surface chemistry, and substrate accessibility prior to experimental implementation.

## Supplementary Material





## Notes

Complete descriptions of system preparation, Go̅Martini parametrization, simulation protocols, analysis scripts, and additional figures (Figures S1–S10) (PDF)
Transparent Peer Review report available (PDF)
