# Supplementary material for: Mechanistic Determinants of Oriented Enzyme Immobilization from Martini Simulations
Source: J Phys Chem Lett. 2026 Feb 9;17(7):2094–102. doi: 10.1021/acs.jpclett.5c03753 (PMC12927024; doi:10.1021/acs.jpclett.5c03753)
Supplement: Supplementary file 1 [file jz5c03753_si_001.pdf]

Supporting Information: Mechanistic Determinants of Oriented Enzyme Immobilization from Martini Simulations

Juan Carlos Jiménez-García,[†,‡] Nicoll Zeballos,[‡] Fernando López-Gallego,[*,‡,¶] Xabier López,[*,†] and David De Sancho[*,†]

†*Polimero eta Material Aurreratuak: Fisika, Kimika eta Teknologia, Kimika Fakultatea, UPV/EHU & Donostia International Physics Center (DIPC), PK 1072, 20018 Donostia-San Sebastian, Euskadi, Spain*

‡*Center for Cooperative Research in Biomaterials (CIC biomaGUNE), Basque Research and Technology Alliance (BRTA), Paseo Miramon 194, 20014 San Sebastián, Spain*

¶*Ikerbasque, Basque Foundation for Science, 48013 Bilbao, Spain*

E-mail: flopez@cicbiomagune.es; xabier.lopez@ehu.eus; david.desancho@ehu.eus

Molecular System Preparation

We focused our study on the tetrameric Alcohol Dehydrogenase (ADH) enzyme from *Bacillus stearothermophilus*, using as a reference the high-resolution crystal structure (PDB ID: 1RJW) reported by Ceccarelli *et al.*[S1] Following the experimental design by Zeballos *et al.*,[S2] three variants were simulated: (i) a wild-type enzyme with an appended N-terminal His-tag modeled in AlphaFold and merged with the crystal structure via UCSF Chimera, and (ii–iii) two engineered histidine cluster variants, H3 (Q8H/K10H/E11H) and H4 (E8H/E11H/E265H/E266H), constructed by introducing the corresponding mutations using the Dunbrack rotamer library.[S3] These engineered variants reproduce the histidine-rich anchoring motifs designed experimentally for site-directed immobilization.

The immobilization support was modelled as a hydrophilic agarose-like surface composed of fixed Martini P4-type beads arranged in a hexagonal lattice with a spacing of 0.47 nm, representing the hydroxyl-rich polysaccharide framework of agarose. The surface dimensions were 12.20 nm $\times$ 11.28 nm and positioned at the base of the simulation box, which was filled with standard Martini3 water beads. No antifreeze particles were required since the Martini3 water model prevents crystallization. Counterions were added to ensure electroneutrality, and each enzyme was initially placed 1.2 nm above the surface and oriented such that the tethering residues (from the His-tag or histidine clusters) faced toward the support.

During immobilization, the enzymes approached and stabilized on the surface through a two-phase process: an initial rapid adsorption followed by tethering-induced equilibration. The adsorption and tethering stages are illustrated in Figure 1, which shows the time evolution of the protein–surface distance and representative snapshots of the approach and stabilized state. Figure S1 shows that, in the absence of a harmonic restraint on the tethering residues, the enzyme undergoes large translational and rotational motions relative to the surface, underscoring the need for a positional anchoring potential to achieve stable immobilization.

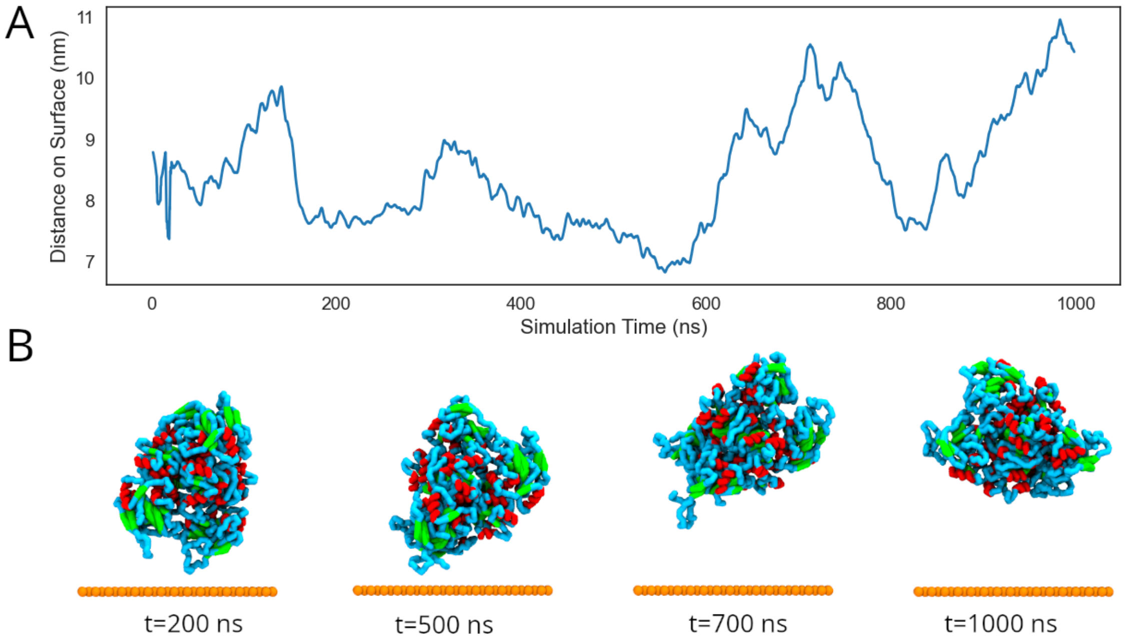

Figure S1: Time evolution of the protein–surface distance during adsorption and representative snapshots of BsADH tethering on the agarose-like surface.

Coarse-Grained Model Generation

The atomic model of ADH was coarse-grained using the Martinize2 pipeline,[S4] producing topologies compatible with the Martini3 force field. Secondary structure restraints were automatically derived from DSSP analysis of the all-atom reference structure to maintain α-helices and β-sheets while allowing flexible loops and termini to remain unconstrained.

Native contact maps were computed once from the experimentally determined all-atom crystal structure of BsADH (PDB ID: 1RJW) using the OV+rCSU algorithm.[S5] In this scheme, native contacts between backbone beads (mapped to C^{α} atoms) are identified by combining geometric overlap of van der Waals volumes (OV) with chemical refinement (rCSU) to define physically meaningful interactions. The resulting contact map was then kept fixed and used as an input for all subsequent GōMartini coarse-grained simulations, following the standard GōMartini protocol. Each native contact was represented by a modified

Lennard–Jones (12–6) potential:

$$V_{\mathrm{LJ}}(r) = 4\varepsilon_{ij}\left[\left(\frac{\sigma_{ij}}{r}\right)^{12} - \left(\frac{\sigma_{ij}}{r}\right)^{6}\right], \tag{1}$$

where $\sigma_{ij} = d/2^{1/6}$ ensures the potential minimum coincides with the native geometry. The depth of the potential was scaled as $\varepsilon_{ij} = \lambda\varepsilon$, with $\varepsilon = 6.276$ kJ/mol and λ controlling native-contact strength. In standard GōMartini implementations, $\lambda = 1.5$ ensures strong stabilization and prevents unfolding below ∼600 K. Here we used $\lambda = 1.2$, which preserves the native fold at 300 K but allows partial unfolding near the experimental melting temperature (T_{m}), providing a balance between structural stability and flexibility. As discussed by Plazinski *et al.*,[S6] such structure-based models intrinsically favor the native state, stabilizing functionally relevant motions while limiting complete unfolding, which suits our focus on immobilization-induced dynamics.

Baseline Conformational Dynamics in Solution

At 300 K, all BsADH variants preserved the native tetrameric structure over 1 µs (Figure S2), with flexibility concentrated in surface loops and terminal regions. $RMSD$ and $RMSF$ analyses confirmed that histidine insertions did not perturb the global fold.

Reducing the native-contact energy to $\lambda = 1.2$ slightly increased backbone fluctuations without destabilizing the fold, as shown in Figure S3. Flexibility remained localized to solvent-exposed loops and helices adjacent to the engineered histidine sites.

Molecular Dynamics Simulations

All simulations were performed with GROMACS 2024, using 10 independent replicas per system and condition. After energy minimization, systems were equilibrated in NVT and NPT ensembles, followed by 1 µs production runs at 300 K and 1 bar using the velocity-rescale thermostat and the Parrinello–Rahman barostat with a 10 fs timestep.

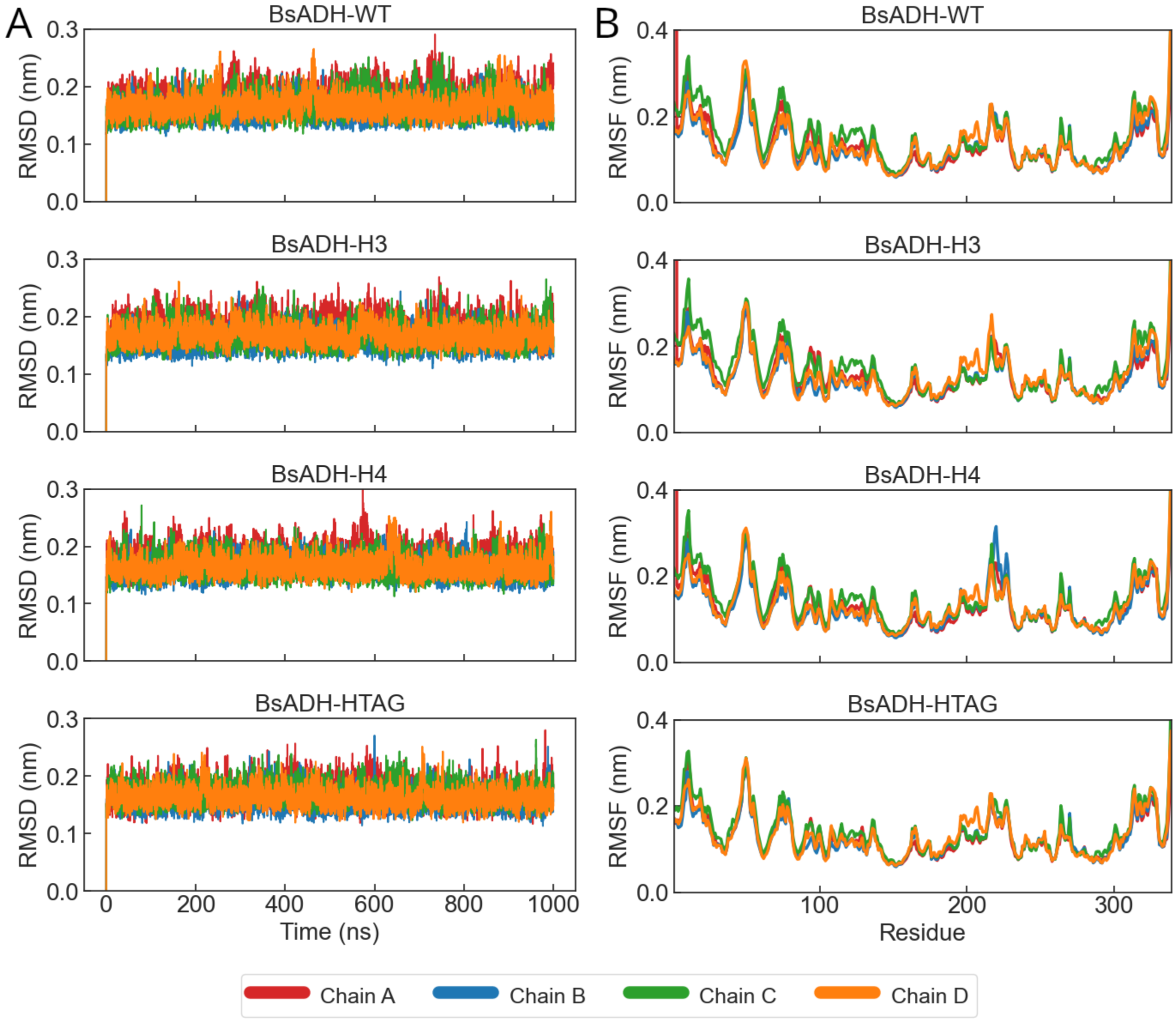

Figure S2: Backbone $RMSD$ (A) and $RMSF$ (B) of BsADH variants in solution over 1 µs. All constructs maintain the native fold, with fluctuations localized to flexible regions.

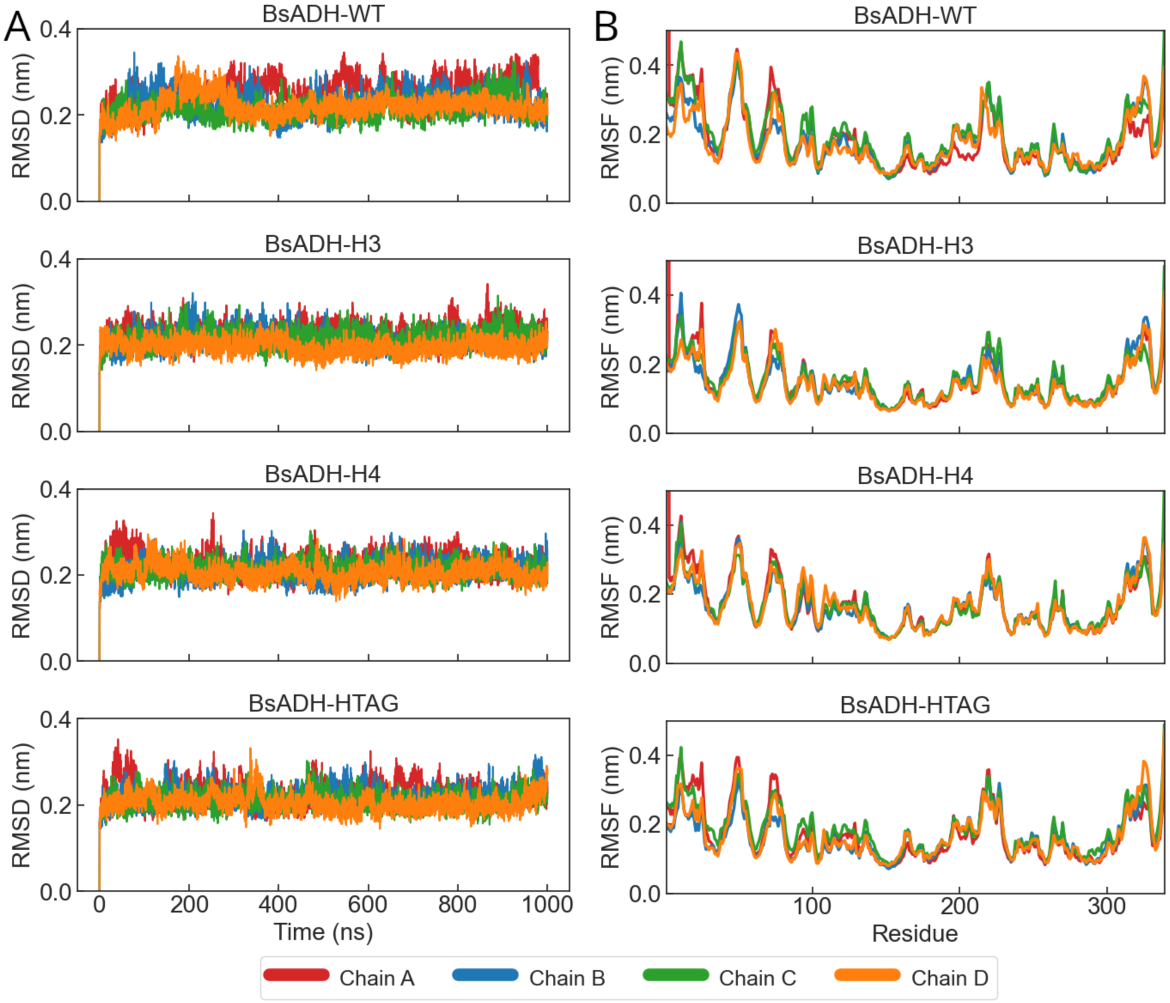

Figure S3: $RMSD$ (A) and $RMSF$ (B) for BsADH variants in solution using $\lambda = 1.2$. Fluctuations increase slightly while preserving the native topology.

The immobilization protocol consisted of two sequential stages. In an initial deposition step, a steered pulling protocol was applied along the surface normal (Z-axis) to promote a rapid and controlled approach of the enzyme toward the surface, using a pulling rate of 0.001 nm ps^{-1} and force constant of 1000 kJ $mol^{-1}nm^{-2}$.

Once the enzyme reached the vicinity of the support, the pulling was switched off and tethering was modeled using static harmonic umbrella restraints. These restraints were applied to the histidine beads involved in immobilization (either terminal His-tags or engineered histidine clusters), while the surface was represented by fixed P4-type beads mimicking an agarose-like support. A force constant of 1000 kJ $mol^{-1}nm^{-2}$ was used to restrain the histidine beads at a target distance of 0.8 nm from the surface, with the pulling rate set to zero.

After equilibration in NVT and NPT ensembles, immobilized systems underwent production simulations in a NPT ensemble, which we split into two phases: an initial deposition stage of 200 ns allowing lateral diffusion, followed by a tethered phase of 1000 ns under full restraint. Soluble controls used identical conditions but without restraints.

Two exceptions were made to this simulation protocol for both soluble and immobilized systems. First, in simulations assessing conformational stability under thermal stress, a modified protocol was applied to prevent numerical instabilities of the Martini waters at high temperatures: following standard NVT and NPT equilibration, production runs were conducted in the NVT ensemble at 300, 400, and 500 K. This adjustment allowed for controlled thermal unfolding while maintaining simulation stability. Second, to evaluate ethanol binding, production simulations were extended to 5 μs to ensure sufficient sampling of transient substrate interactions.

Conformational Dynamics upon Immobilization

$RMSD$ and $RMSF$ profiles (Figure S4) reveal reduced flexibility in tethered chains compared with solvent-exposed subunits, reflecting the spatial constraints imposed by surface an-

choring. Free-energy landscapes derived from the native-contact fraction Q (Figures S5–S7) show that immobilized variants retain higher Q values and deeper minima across temperatures, consistent with enhanced structural stability.

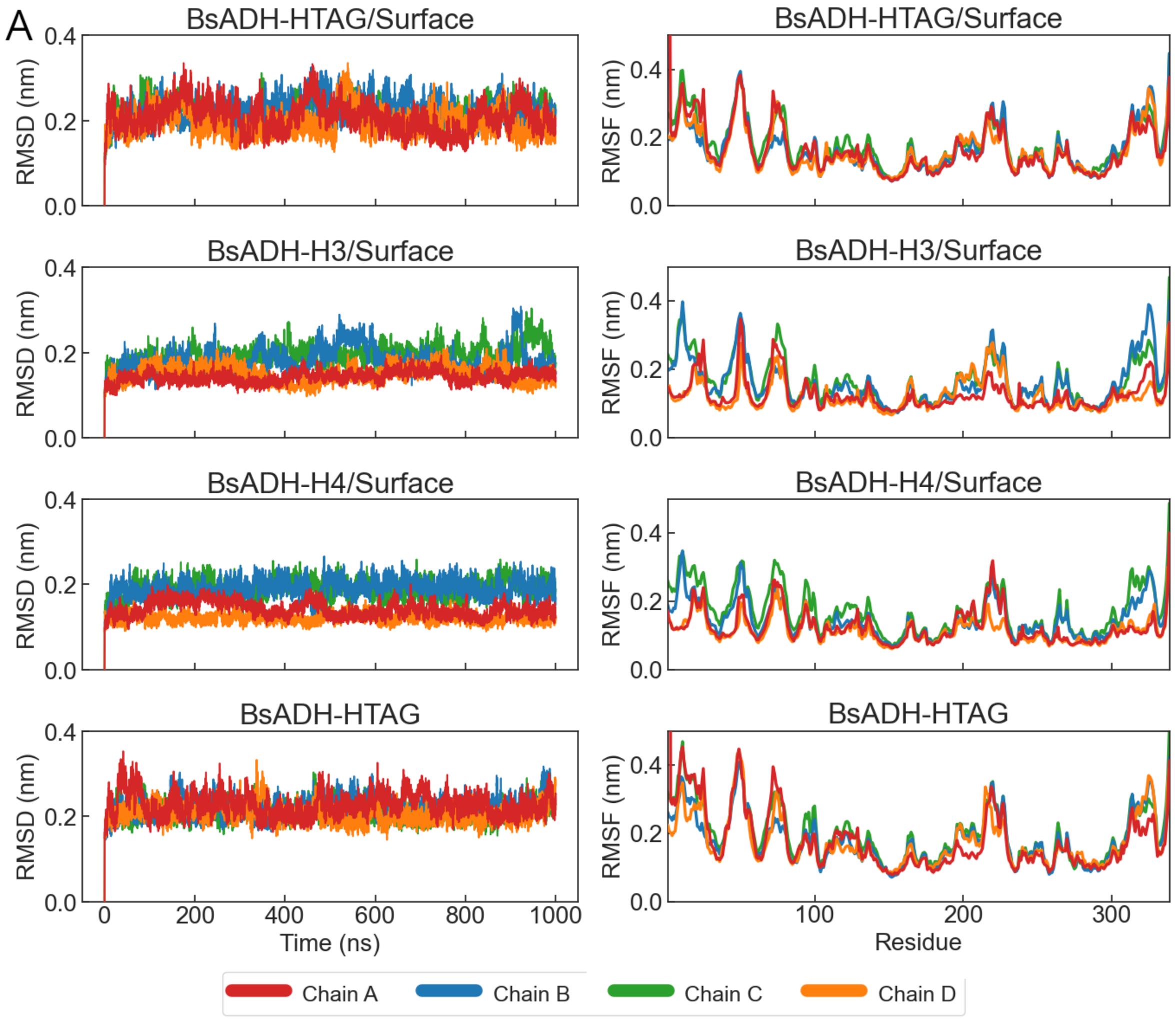

Figure S4: $RMSD$ (A) and $RMSF$ (B) for immobilized BsADH variants ($\lambda = 1.2$). Tethering locally restricts motions of surface-contacted subunits.

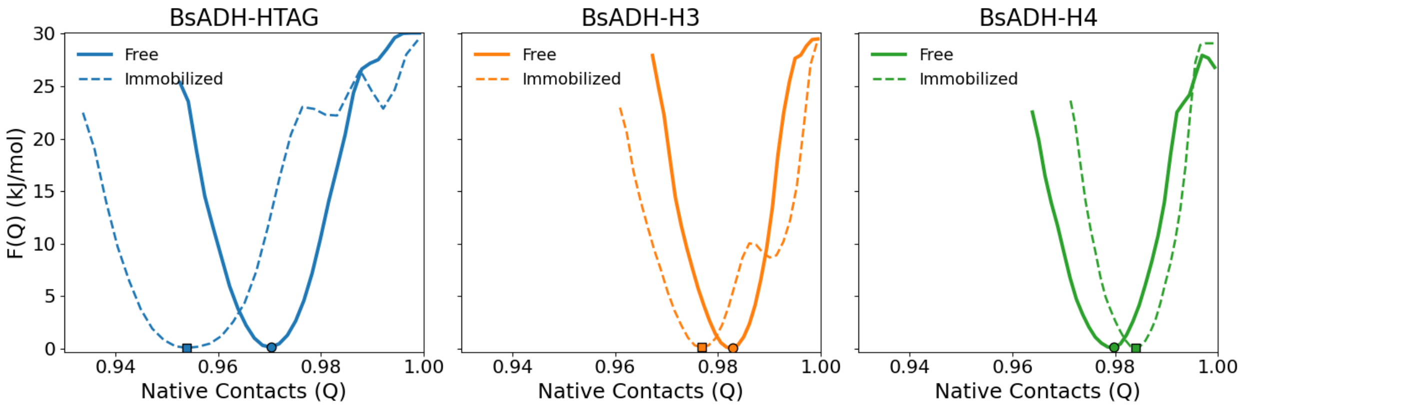

Figure S5: Free-energy landscape $F(Q)$ for soluble (solid) and immobilized (dashed) BsADH variants at 300 K.

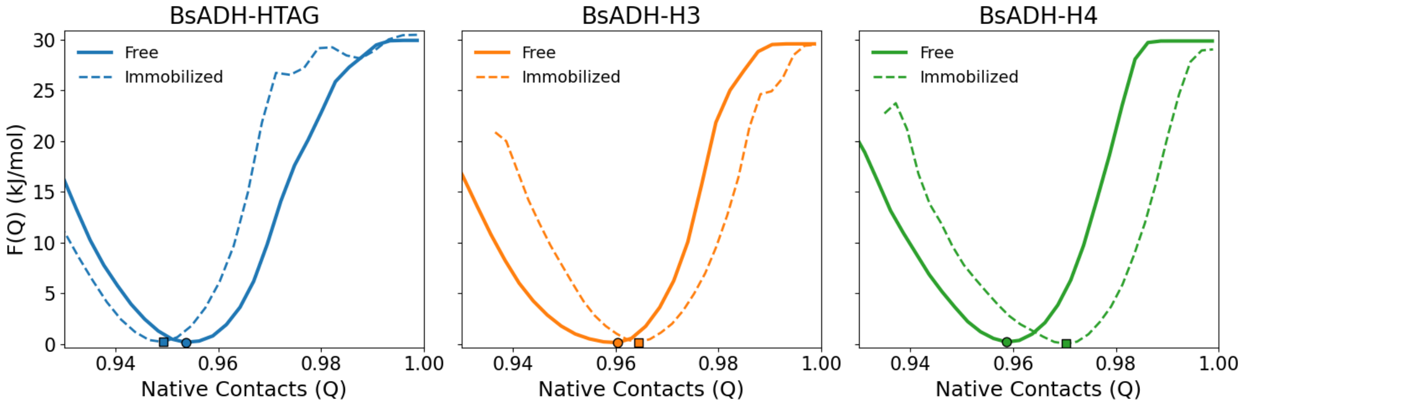

Figure S6: Free-energy landscape $F(Q)$ at 400 K showing moderate destabilization in soluble forms and higher resilience upon immobilization.

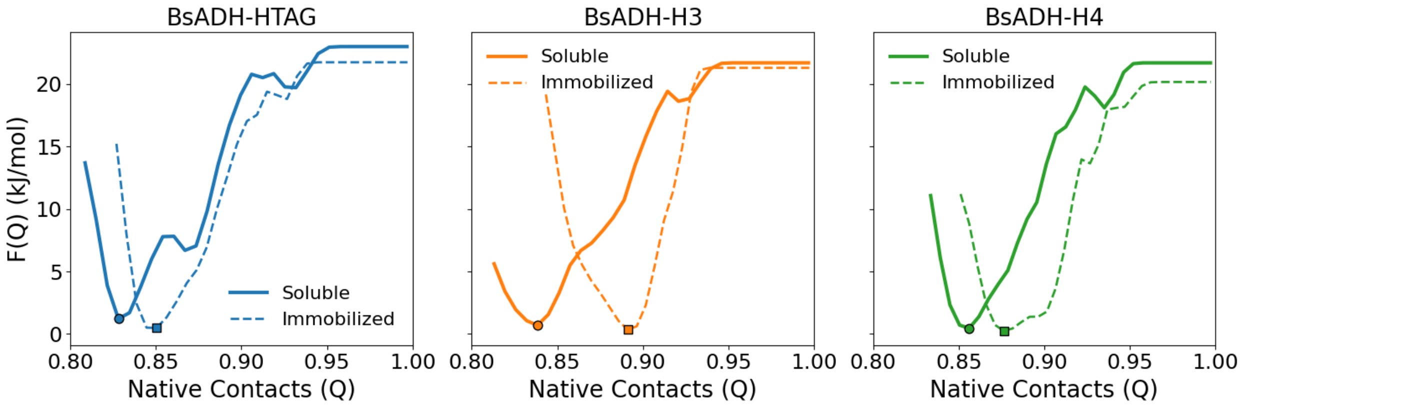

Figure S7: Free-energy landscape $F(Q)$ at 500 K revealing partial unfolding in soluble enzymes and higher structural retention in immobilized variants.

Functional Analysis: Substrate Binding and Cofactor Release

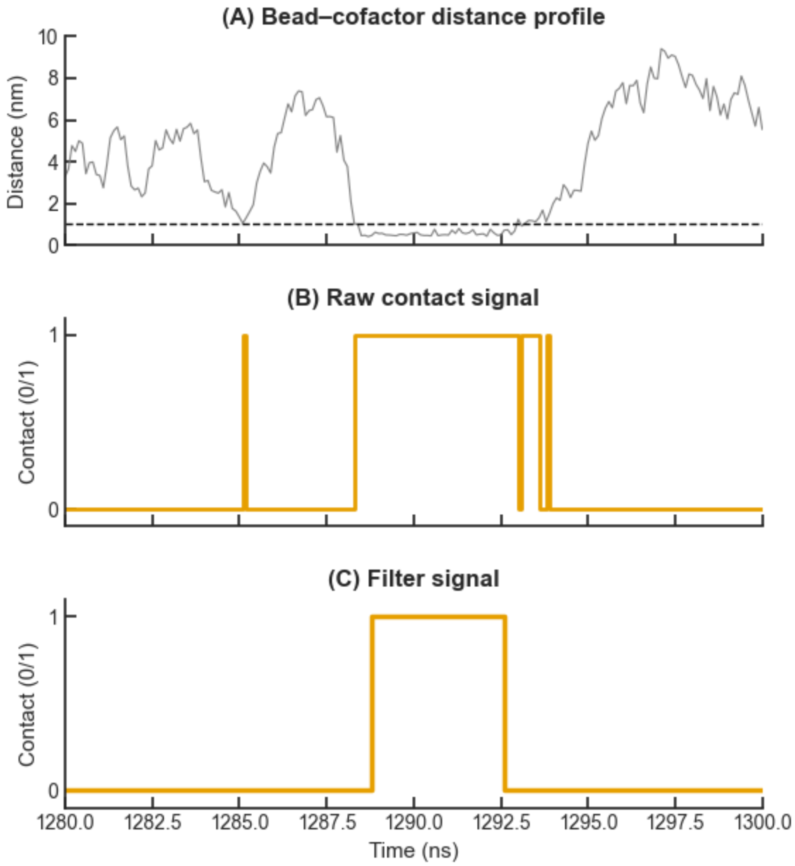

Figure S8: Schematic representation of the procedure used to identify contact events between ethanol beads and the cofactor. (A) Time evolution of the bead–cofactor distance. (B) Binary contact signal obtained directly from the distance threshold (<0.8 nm), showing short transient spikes due to noise. (C) Filter signal obtained by applying a moving average filter (window = 10), which removes spurious spikes and retains persistent binding events.

To assess functional effects, ethanol binding was analyzed in NAD^+-bound systems, with NAD^+ positioned by aligning 1RJW with the NAD-bound crystal 3JV7.[S7] Ethanol and NAD^+ parameters followed Barriga-Alves *et al.*[S8] Ethanol–protein proximity dynamics were evaluated through a distance-based contact analysis (Figure S8). For each trajectory, the minimum distance between ethanol beads and the cofactor was monitored over time to detect approach events. Distances shorter than 0.7 nm were classified as contacts, generating a binary signal (0/1) that reports the presence or absence of interaction at each frame. Since the raw contact signal can include high-frequency spikes corresponding to transient or noisy

collisions, a moving average smoothing filter (window = 10 frames) was applied to retain only sustained contact periods. This filtered signal was then used to identify and quantify individual binding events, from which the contact duration and frequency were computed for each system.

Cofactor (NADH) dissociation kinetics (Figure S9) revealed that immobilization generally increases NADH residence times, though the cluster variants show milder slowdowns compared to HTAG. The mean first-passage time (τ_{ML}) was estimated using a maximum-likelihood approach:

$$\tau_{\mathrm{ML}} = \frac{1}{N_d}\left(\sum_{i=1}^{N_d} t_i + \sum_{j=1}^{N-N_d} t_j\right),$$

with standard error $\mathrm{SE} = \tau_{\mathrm{ML}}/\sqrt{N_f}$.

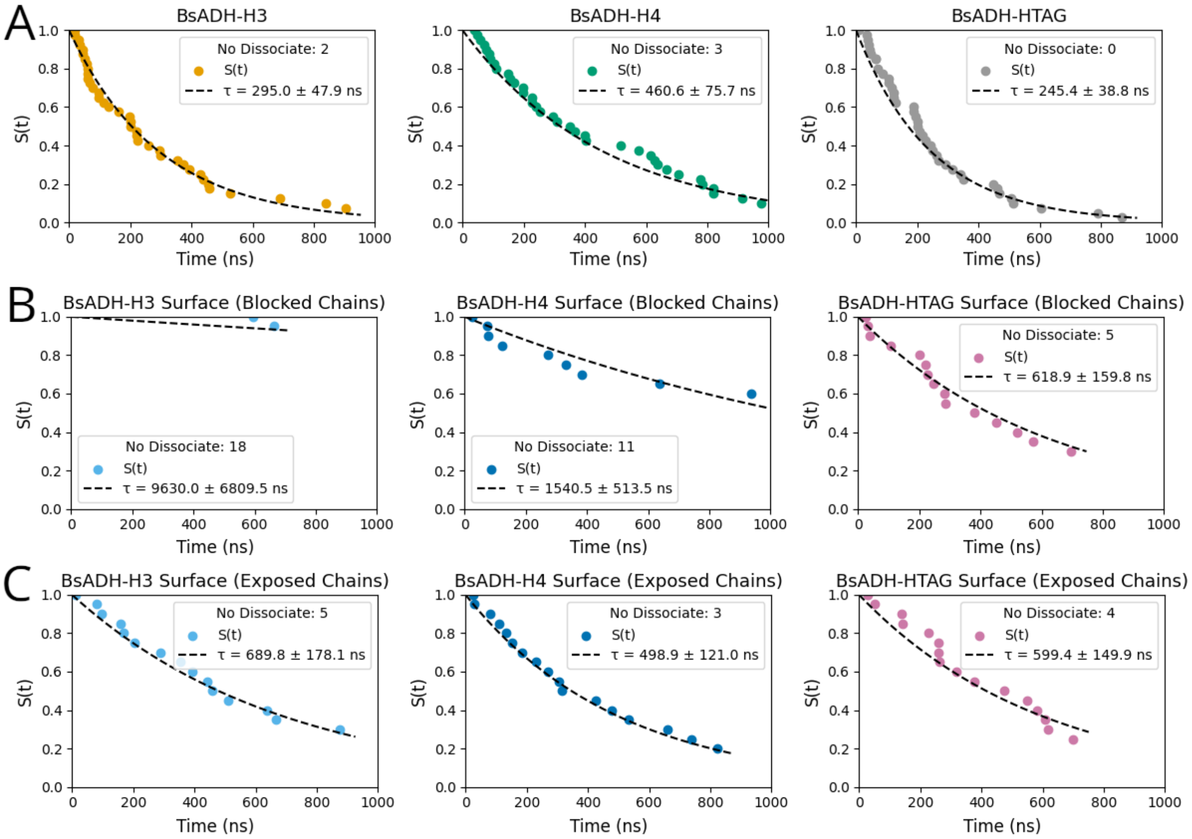

Figure S9: NADH residence and dissociation in BsADH: (A) soluble enzymes, (B) immobilized variants, and (C) chain-specific analysis. Surface tethering increases residence times, with H3/H4 showing milder effects than HTAG.

Experimental Materials and Methods

Materials

Cobalt-activated agarose microbeads 6BCL were acquired from ABT Technologies (Madrid, Spain). Isopropyl-β-D-thiogalactopyranoside (IPTG) was obtained from Gerbu Biotechnik GmbH (Wieblingen, Germany). SYPRO® Orange Protein Gel Stain was purchased from Sigma-Aldrich (St. Louis, IL, USA). Bradford protein assay dye reagent and Micro Bio-Spin columns were purchased from Bio-Rad (Hercules, CA, USA).

Protein Expression and Purification

Enzymes were overexpressed as previously described by Zeballos *et al.* Briefly, plasmids encoding each enzyme were transformed into chemically competent *E. coli.* Single colonies were used to inoculate 3 mL of LB medium supplemented with 30 $\mu g\,mL^{-1}$ kanamycin and incubated overnight at 37 °C.

Then, 50 mL of LB medium containing the same antibiotic concentration were inoculated with 1 mL of the overnight culture in a 250 mL flask and grown at 37 °C and 250 rpm until reaching an optical density of $OD_{600} = 0.6$.

Protein expression was induced with:

- 1 mM IPTG for BsADH-HTAG (3 h at 37 °C, 250 rpm)
- 0.1 mM IPTG for BsADH-H3 (overnight at 21 °C, 250 rpm)

Cell pellets were resuspended in 25 mM Tris-HCl buffer (pH 7.0) containing 150 mM NaCl and 10 mM imidazole. Cells were lysed by sonication (Bandelin Somoplus sonicator) on ice for 20 min (40% amplitude, 5 s pulses). The lysates were centrifuged at 9391 g for 30 min at 4 °C to recover the soluble protein fraction.

Enzymes were immobilized on cobalt-chelate functionalized resins. A volume of 5 mL of crude extract was incubated with 500 mg of resin under gentle stirring for 1 h. Columns

were washed three times with 25 mM Tris-HCl buffer (pH 7.0), and the immobilized enzymes were stored at 4 °C until use.

Differential Scanning Fluorimetry (DSF)

Differential scanning fluorimetry (DSF) was performed using 8 µg of each immobilized enzyme. Experiments were conducted using a Bio-Rad CFX Connect™ Real-Time Detection System.

A final volume of 20 µL was prepared in a 200 µL PCR tube containing the immobilized enzyme and 2.5 µL of SYPRO® Orange Protein Gel Stain (40-fold diluted from stock). Samples were heated from 25 °C to 95 °C at a rate of 0.5 °C min^{-1}.

The first derivative of the fluorescence curve (dF/dT) was plotted to determine the melting temperature (T_M) corresponding to the maximum of the derivative curve (Figure S10).

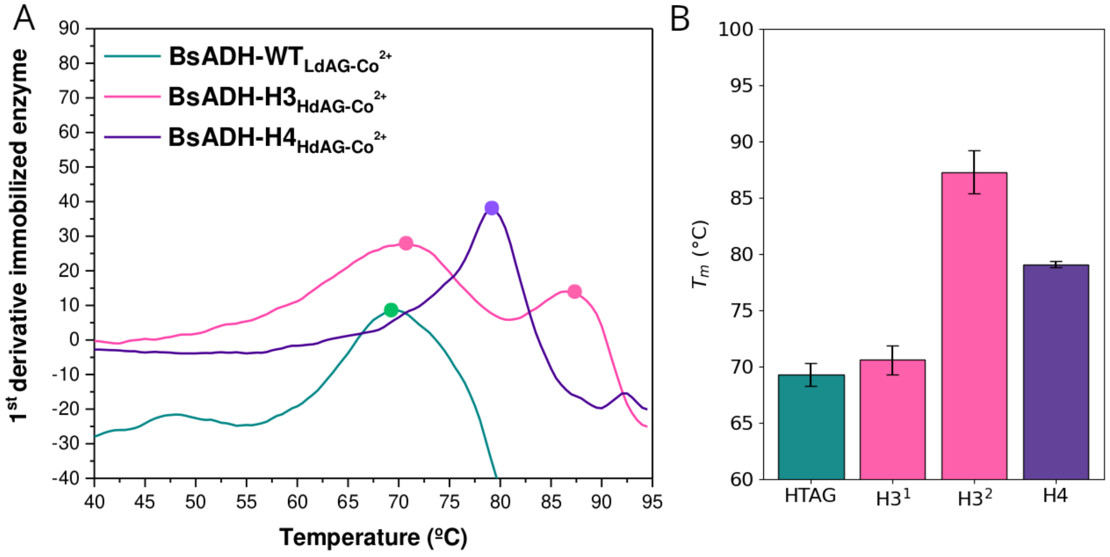

Figure S10: **(A)** Dynamic scanning fluorimetry plots of the different BsADH variants immobilized on agarose-based microbeads functionalized with cobalt-chelates. **(B)** Melting temperature (T_m) values of immobilized BsADH variants: BsADH-WT$_{LdAG-Co^{2+}}$, 69.3 ± 1.0 °C; BsADH-H3$_{HdAG-Co^{2+}}$ exhibits two transitions at 70.6 ± 1.3 °C and 87.3 ± 1.9 °C; and BsADH-H4$_{HdAG-Co^{2+}}$, 79.1 ± 0.29 °C.

References

(S1) Ceccarelli, C.; Liang, Z.-X.; Strickler, M.; Prehna, G.; Goldstein, B. M.; Klinman, J. P.; Bahnson, B. J. Crystal structure and amide H/D exchange of binary complexes of alcohol dehydrogenase from Bacillus stearothermophilus: insight into thermostability and cofactor binding. *Biochemistry* **2004**, *43*, 5266–5277.

(S2) Zeballos, N.; Comino, N.; Sanz, D. A.; Santiago-Arcos, J.; Azkargorta, M.; Elortza, F.; Diamanti, E.; López-Gallego, F. Region-directed enzyme immobilization through engineering protein surface with histidine clusters. *ACS Appl. Mater. Interfaces* **2023**,

(S3) Shapovalov, M. V.; Dunbrack, R. L. A smoothed backbone-dependent rotamer library for proteins derived from adaptive kernel density estimates and regressions. *Structure* **2011**, *19*, 844–858.

(S4) Souza, P. C. T.; Alessandri, R.; Barnoud, J.; Thallmair, S.; Faustino, I.; Grünewald, F.; Patmanidis, I.; Abdizadeh, H.; Bruininks, B. M. H.; Wassenaar, T. A.; others Martini 3: a general purpose force field for coarse-grained molecular dynamics. *Nat. Methods* **2021**, *18*, 382–388.

(S5) Poma, A. B.; Cieplak, M.; Theodorakis, P. E. Combining the MARTINI and structure-based coarse-grained approaches for the molecular dynamics studies of conformational transitions in proteins. *J. Chem. Theory Comput.* **2017**, *13*, 1366–1374.

(S6) Plazinski, W.; Lutsyk, V.; Plazinska, A. Exploring free energies of specific protein conformations using the Martini force field. *J. Chem. Theory Comput.* **2024**, *20*, 2273–2283.

(S7) Karabec, M.; Łyskowski, A.; Tauber, K. C.; Steinkellner, G.; Kroutil, W.; Grogan, G.; Gruber, K. Structural insights into substrate specificity and solvent tolerance in alcohol dehydrogenase ADH-'A' from Rhodococcus ruber DSM 44541. *Chem. Commun.* **2010**, *46*, 6314–6316.

(S8) Barriga, R. M. d. S. A. Simulating substrate binding sites in the *S. aureus* Type II NADH Dehydrogenase. Master's thesis, Universidade Nova de Lisboa, Instituto de Tecnologia Química e Biológica António Xavier, Oeiras, Portugal, 2022; Supervisor: Manuel N. Melo; co-supervisor: Manuela M. Pereira.
