# Supplementary material for: Mechanistic Determinants of Oriented Enzyme Immobilization from Martini Simulations
Source: J Phys Chem Lett. 2026 Feb 9;17(7):2094–102. doi: 10.1021/acs.jpclett.5c03753 (PMC12927024; doi:10.1021/acs.jpclett.5c03753)
Supplement: Supplementary file 2 [file jz5c03753_si_002.pdf]

jz-2025-03753z.R1

Name: Peer Review Information for "Mechanistic Determinants of Oriented Enzyme Immobilization from Martini Simulations"

First Round of Reviewer Comments

Reviewer: 1

Comments to the Author

In my opinion, the authors try to make a modelling on the enzyme immobilization effects, but in this way, some simplifications are taken. They use a support that may have several active groups below each enzyme molecule (more considering the very large size of this enzyme) and the interaction of these groups with the enzyme are not considered (metal or even carboxylic groups if the metal is lost). Perhaps using a tailor-make support offering just on metal chelate under each enzyme molecule and then increasing the number of active groups on the support, the conclusions could become more accurate and supported. The exact geometry of the support and distribution of the active groups is unknown. They do not consider the practical usefulness of this methodology. Co2+ may act as a catalysts, the enzyme support interaction is weak and the enzyme can move, the metal may be released and the enzyme can be released, becoming a no very useful immobilization protocol. They try to give to closed conclusions based in many measurements but ignoring all likely interferences.

Minor points to be also considered may be found below:

Abstract do not offer any data on recovered activity nor immobilization yield. Neither the support nor the active groups in the support are mentioned. Results are just qualitative statements. Please, rewrite the abstract giving main hypothesis, results (quantitative) and conclusions.

“while preserving the mobility of exposed regions, resulting in enhanced conformational stability under thermal stress.” Looks contradictory.

Introduction. If they want to list the uses of enzymes, energy and analytical chemistry should be mentioned. However, I suggest better to eliminate the specific mention of uses

of enzymes and leave that in a more general way, giving some references on reviews on enzymes in green chemistry.

Please, enrich the introduction of immobilization. It started as a way to recover and reuse these initially very expensive biocatalysts, now it have shown to be a ppotent tool to improve multiple enzyme features: stability is the usual target, this may be achieved very different causes: multipoint or multisubunit immobilization, generation of favorable enzyme environments, etc. There is some reviews that could be useful in this point. Moreover, this makes the operation window of the biocatalysts greater. The conformation changes that immobilization produce can led to tuning enzyme selectivity, specificity, inhibition, etc. Furthermore, the enzyme purification can be coupled to the enzyme immobilization. Again, there are reviews on almost each of these topics.

"As a result, enzymes are mainly immobilized through random orientation, yielding unproductive enzymes with either unfavorable orientations or excessively rigid conformations." Really, it has been shown that some of the most popular immobilization techniques produce an enzyme orientation, although it may be no easy to know which are the involved areas, that is, uncontrolled orientation may be a better way to define this than random (although in some instances it is true that may be uncontrolled and random). What the authors means but "unfavorable orientation"? What mean by excessive rigidification? Many authors pursue a maximum multipoint covalent attachment and in many instances enzyme activity is fairly maintained. Uncontrolled enzyme-support interactions may led distortion and this to enzyme activity decrease. Please, explain much better, using adequate references.

"However, the orientation through the His-tag is not always optimal to maximize the activity/stability balance as it can trigger an inactive conformation when bound to Surface" Explain this in more detail, at first glance I disagree with this.

The use of areas of proteins bearing several His has been proposed by other authors, the idea is not fully new. In any case, they need to explain before the mechanism of enzyme immobilization on immobilized metals and why this can direct the enzyme immobilization. However, this cannot prevent further enzyme-support interactions between the support and the enzyme surface, as the enzyme groups are very near to the support groups. Authors have shown in previous papers how enzyme can move when immobilized on IMAC, why this is not considered here?

Using a tetraedric enzymes, they have 4 areas rich in His, making all more complex to control.

Reviewer: 2

Comments to the Author

This is a very interesting paper focusing on the issue of modeling the process of immobilizing protein enzymes on surfaces at a coarse-grained resolution, as well as the structural consequences of this process that determine enzyme activity. The described computational methodology itself is novel, albeit somewhat intuitive. However, the value of the paper is elevated by the following factors: (1) the thoroughness and systematic approach to the analysis, and (2) the multifaceted view of the problem, which goes beyond just conformational analysis and also includes indirect descriptions of enzymatic activity.

* The paper would benefit from a more detailed description of the experimental research section, especially its relevance in the context of simulation-based studies (which occupy the majority of the paper), as well as the significance of the results obtained. Currently, the information from the experimental part is almost absent in the main manuscript, and its existence is primarily indicated in the appropriate section in the Supplementary Information.

* The weak point of the study, in my opinion, is the assumption of an ideally flat agarose surface. In reality, the agarose surface may be porous and contain various partially detached polysaccharide chains. The model adopted in the present work (the hexagonally arranged surface of the P4 Martini beads) is certainly a simplification. That said, I realize that creating a more realistic model may be challenging due to limited structural information. On the other hand, saccharide-specific parameters within Martini 3 exist, which makes it at least technically feasible. I suggest that the authors discuss the limitations of the model arising from this assumption.

* This issue also has another aspect: would the procedure proposed by the authors be technically feasible for a different surface exhibiting different properties, such as charged saccharides, mineral materials, etc.? To what extent is the approximation of a flat, homogeneous surface adequate for this type of study?

* Is it possible that the lack of spontaneous enzyme adsorption on the surface (Fig. S1) is due to the absence of mobile anchors (polysaccharide chains) or porous surfaces, which would facilitate adsorption in realistic systems through their geometry?

* The authors use the GoMartini approach to simulate the higher-order structure of proteins, which is a correct choice given the possible conformational changes induced by surface binding or temperature fluctuations. Do the authors believe that an alternative approach, such as using an elastic network model, would yield qualitatively consistent results with those described in the paper? Were such simulations carried out, for instance, in preliminary studies?

Minor Points:

* Missing figure number in SI (Fig. ??).

* Some details regarding the pulling protocol may be worth mentioning: To which beads was the pulling force applied? What were the pulling rate and force constants in the pulling potential?

Reviewer: 3

Comments to the Author

The work presented by Jimenez-Garcia et al. employs the widely used coarse-grained Martini 3 model to study conformational changes in protein systems. The study is novel and valuable, with potential for broad application across bioengineering and related fields, including those with more industrially oriented objectives. The authors have conducted and selected the methods that best approximate the problem under study. Moreover,

structural and kinetic differences determined by MD simulations, among the systems analyzed, are clearly evidenced. Some comments that could help improve the manuscript are provided below.

Main comments:

1) In Figure 1A, the structure could be better represented in cartoon format, with secondary structure elements labeled.

2) In Figure 1B, the chōon (ō) symbol should be used for GōMartini and other instances.

3) Could the authors specify how the contact map was determined in the study. Typically, the contact map (OV+rCSU) in GōMartini simulations is computed from an experimentally derived structure and then used for CG simulations.

4) What are the limitations of considering the Martini P4-type as agarose-like? Additionally, later in paragraph 8 it is mentioned that anchoring without restraints does not show a stable interaction (protein/support). Indeed, the strategy used is interesting for modeling this type of system; however, it is important to clearly state the limitations of the model in terms of protein–surface interactions.

5) In Figure 2B, since a frame at t = 50 ns is shown, it would be convenient to indicate in panel A the moment at which this transition occurs.

6) The GōMartini approach can have limitations regarding conformational sampling in multi-chain systems. In this case, the high stability observed could be due to an artifact resulting from fa high number of contacts in the contact map. This could be discussed to highlight the limitations of the model.

7) It would be easier for the reader to identify the A and D subunits in Figures 3C–E. Moreover, are these letters (A–D) corresponding to domain names or only to chain IDs?

8) Figure 3 could benefit from a SASA analysis of the whole system, comparing the His-tagged construct and the variants.

9) On page 4, in the third paragraph, the respective EtOH concentration could be indicated.

10) The authors mention that the modeling of EtOH binding and NADH release is well correlated with experimental data. Figure 5B could include experimental k_on values as a reference.

11) The aforementioned results are interesting. I would therefore recommend describing geometric parameters of the catalytic binding site, such as SASA or volume as a function of time.

Author's Response to Peer Review Comments:

Reviewer: 1

Recommendation: This paper is not recommended because it does not provide new physical insights.

We thank the referee for his/her detailed comments on the manuscript, which we address fully in our response below.

Comments:
In my opinion, the authors try to make a modelling on the enzyme immobilization effects, but in this way, some simplifications are taken. They use a support that may have several active groups below each enzyme molecule (more considering the very large size of this enzyme) and the interaction of these groups with the enzyme are not considered (metal or even carboxylic groups if the metal is lost). Perhaps using a tailor-made support offering just on metal chelate under each enzyme molecule and then increasing the number of active groups on the support, the conclusions could become more accurate and supported. The exact geometry of the support and distribution of the active groups is unknown. They do not consider the practical usefulness of this methodology. Co2+ may act as a catalysts, the enzyme support interaction is weak and the enzyme can move, the metal may be released and the enzyme can be released, becoming a no very useful immobilization protocol. They try to give to closed conclusions based in many measurements but ignoring all likely interferences.

Response: We agree that our modeled surface relies on several assumptions and simplifications. However, we respectfully disagree with some of the arguments raised by Reviewer 1.

First, in our system, each enzyme is anchored to the surface through multiple attachment points. Specifically, the clustered variants are immobilized via two or three anchoring points involving different subunits, since coordination to the cobalt chelate requires the simultaneous interaction of two or three His residues. For all the variants studied here, anchoring occurs through two or three points distributed across two different subunits

(see Figure 3C-E). Accordingly, our model explicitly considers the presence of multiple anchoring points beneath each tetramer.

Based on the functionalization degree of the 6BCL agarose beads (15 μmol g^{-1}) and their surface area (26 m^2 g^{-1}), the estimated surface density of cobalt chelates is approximately 0.35 chelate groups per nm^2. Considering that the estimated footprint of the BsADH tetramer from its X-ray crystal structure is ~100 nm^2, we estimate that approximately 35 chelate groups are present beneath each enzyme, thus ensuring multivalent attachment.

Importantly, this chelate density is 5,285-fold lower than the density of the D-galactose and 3,6-anhydro-L-galactose monomers (agarobiose repeating unit; Mw = 324.28 g mol^{-1}) composing the agarose matrix, which corresponds to ~1,850 agarobiose units per nm^2. Based on this comparison, we are confident that the overall physicochemical properties of the surface in contact with the enzyme are dominated by the saccharide groups rather than by the cobalt chelates. As Reviewer 2 suggests, unreacted metal chelates could potentially interact nonspecifically with the enzyme surface; however, the overwhelming excess of agarobiose units relative to cobalt chelates strongly suggests that nonspecific enzyme-support interactions are primarily governed by the hydroxyl-rich saccharide matrix.

Thus, cobalt chelates mainly serve to direct immobilization through the His-enriched regions, as captured by our simulations, while the broader enzyme-support interactions are dictated by the agarose surface. Hence, we are confident that P4-type surface is a good approximation to the surface of agarose beads. On the other hand, as correctly pointed out by Reviewer 2, the exact spatial distribution of cobalt chelates on the surface of porous agarose beads is unknown and therefore cannot be explicitly modeled. For this reason, we adopted the simplified representation of a uniform P4-type surface arranged in a hexagonal lattice with a spacing of 0.47 nm.

Finally, we respectfully disagree with Reviewer 1 regarding the potential role of cobalt in enzyme catalysis. Immobilized metal affinity chemistry has been extensively used for the preparation of heterogeneous biocatalysts and is widely covered in immobilization reviews published over the years (see references 25 and 26 in the revised manuscript). This approach has been applied to a broad range of enzymes, with numerous studies reporting minimal interference with catalytic mechanisms due to the presence or potential leaching of metal ions. While this immobilization strategy is reversible, the low dissociation constant (in the low micromolar range) ensures stable immobilization under mild conditions and in the absence of competitors (e.g., imidazole). Moreover, this methodology has been successfully applied to oxidoreductases, transferases,

hydrolases, lyases, and ligases, as well as to the co-immobilization of multi-enzyme systems composed of His-tagged enzymes (e.g., Grajales-Hernández et al, *ACS Catal*.l, 2023 DOI: 10.1021/acscatal.3c02615). The potential shortcomings mentioned by Reviewer 1 are therefore unlikely to be relevant under the reaction conditions used to assay BsADH (neutral pH, aqueous media, 30 °C).

Minor points to be also considered may be found below:

Abstract does not offer any data on recovered activity nor immobilization yield. Neither the support nor the active groups in the support are mentioned. Results are just qualitative statements. Please, rewrite the abstract giving the main hypothesis, results (quantitative) and conclusions.

Response: We have not included any experimental data because this paper used already published data to validate the model. The core of this work is developing the coarse-grained model to recapitulate enzyme immobilization with a well-characterized example. We selected a previous work by two of us to validate the model, thus including experimental data in the abstract may be misleading.

“while preserving the mobility of exposed regions, resulting in enhanced conformational stability under thermal stress.” Looks contradictory.

Response: In our opinion, this sentence is not contradictory. We mean that immobilization promotes the rigidification of some regions, which may be starting points for unfolding, leaving other regions flexible enough to play their role in catalysis. Nonetheless, to avoid any misunderstanding, we rephrased this sentence as follows:

> *“We found that cluster-based immobilization locally restricts flexibility in surface-contacting subunits while preserving the mobility of exposed regions, resulting in enhanced conformational stability under thermal stress.”*

Introduction. If they want to list the uses of enzymes, energy and analytical chemistry should be mentioned. However, I suggest better to eliminate the specific mention of uses of enzymes and leave that in a more general way, giving some references on reviews on enzymes in green chemistry.

Response: We have added the applications suggested by the Reviewer 1. The modified sentence in the introduction reads as follows:

“They are used in industrial sectors such as pharmaceuticals, food processing, fine chemical production, energy and sensing[1-7].”

References 1 to 7 also review the newly added applications.

Please, enrich the introduction of immobilization. It started as a way to recover and reuse these initially very expensive biocatalysts, now it have shown to be a potent tool to improve multiple enzyme features: stability is the usual target, this may be achieved very different causes: multipoint or multisubunit immobilization, generation of favorable enzyme environments, etc. There is some reviews that could be useful in this point. Moreover, this makes the operation window of the biocatalysts greater. The conformation changes that immobilization produce can led to tuning enzyme selectivity, specificity, inhibition, etc. Furthermore, the enzyme purification can be coupled to the enzyme immobilization. Again, there are reviews on almost each of these topics.

Response: In our opinion, the benefits of enzyme immobilization have been briefly described in our introduction, directing the readers to some outstanding examples and seminal reviews in the field for more details (see ref 8-18). All the topics highlighted by Reviewer 1 were already mentioned in our original introduction. Please, read paragraph 1 of page 1.

“As a result, enzymes are mainly immobilized through random orientation, yielding unproductive enzymes with either unfavorable orientations or excessively rigid conformations.” Really, it has been shown that some of the most popular immobilization techniques produce an enzyme orientation, although it may be no easy to know which are the involved areas, that is, uncontrolled orientation may be a better way to define this than random (although in some instances it is true that may be uncontrolled and random). What the authors means but “unfavorable orientation”? What mean by excessive rigidification? Many authors pursue a maximum multipoint covalent attachment and in many instances enzyme activity is fairly maintained. Uncontrolled enzyme-support interactions may led distortion and this to enzyme activity decrease. Please, explain much better, using adequate references.

Response: Following this suggestion, we replaced “random” by “uncontrolled”. It is true that many immobilization chemistries may exhibit some regiospecificity for certain surface regions. However, as Reviewer 1 points out, it is very difficult to infer what is the most populated orientation in typical immobilization protocols.

Unfavourable orientation means orientation where either the enzyme activity or stability is decreased upon immobilization. An excessive rigidification means that the immobilization can rigidify certain regions to some extent, where other regions required for the catalysis lose flexibility, negatively impacting enzyme performance.

As widely reported in literature, when enzymes are immobilized, we face the activity-stability trade-off (see Bisirri et al, *Biomacromolecules*, 2023, DOI: acs.biomac.3c00396; and ref 68). This is the reason why a very intense multivalent attachment through uncontrolled interactions may distort the enzyme structure, diminishing its catalytic activity, but make the enzyme more robust to environmental changes (e.g high temperature, drastic pH, organic cosolvents….)

"However, the orientation through the His-tag is not always optimal to maximize the activity/stability balance as it can trigger an inactive conformation when bound to Surface" Explain this in more detail, at first glance I disagree with this.

Response: This is the typical case when the N- or C-termini of a given enzyme play a role in catalysis. Therefore, the enzyme immobilization through either termini is negative for the catalytic efficiency. We have further explained this concept through adding the following sentence:

> *"This case is illustrated with enzymes where their N- or C-termini play a role in catalysis."*

The use of areas of proteins bearing several His has been proposed by other authors, the idea is not fully new. In any case, they need to explain before the mechanism of enzyme immobilization on immobilized metals and why this can direct the enzyme immobilization. However, this cannot prevent further enzyme-support interactions between the support and the enzyme surface, as the enzyme groups are very near to the support groups. Authors have shown in previous papers how enzyme can move when immobilized on IMAC, why this is not considered here?

Response: We agree with Reviewer 1 that directed immobilization through His-clusters has already been reported by some of us in previous articles. However, this paper does not aim to report this mechanism; what this work aims to disseminate is the model that can help us to understand the changes in functionality and stability once a given enzyme is immobilized through a known region. As His-driven immobilization is extremely selective, we selected this system to validate our model. Our model does not predict the orientation; this is given by us based on the immobilization selectivity; instead, it sheds

light on the structural rearrangements and substrate/cofactor interactions once the enzyme is immobilized on the given orientation. The model results are consistent with the experimental findings.

Regarding the last comment on this point, we indeed considered our previous paper since the work published by Zeballos et al (Ref 40) experimentally describes the system herein presented as stated in the introduction (Page 2). We have extended the following sentence for greater clarity:

> "*In Figure 1A we show a representation of the crystal structure of BsADH,*[61] *which we modelled in soluble and immobilized forms following the system reported by Zeballos et al.*[40] *In the experiments, different variants of BsADH containing either a N-terminus His-tag or His-cluster at different enzyme regions were immobilized on porous agarose beads functionalized with metal chelates through affinity guided immobilization based on metal-imidazol coordination bonds between the enzyme and the support surfaces*".

Using tetraedric enzymes, they have 4 areas rich in His, making all more complex to control.

Response: Reviewer 1 is correct in noting that BsADH contains four His-enriched regions in all variants, including both His-tagged and His-clustered forms. However, due to the symmetry of the tetramer, immobilization is restricted to two subunits at a time.

Consequently, only two of the four subunits directly interact with the support surface. Importantly, the two possible interaction planes are symmetrical, which effectively results in a single immobilization orientation, as illustrated in Figure 1E and Figure 3C–E.

Although this system is indeed more complex to control, as pointed out by Reviewer 1, we believe that validating our model using a multimeric enzyme is justified, given that most enzymes are multimeric in nature.

Reviewer: 2

Recommendation: This paper is publishable subject to minor revisions noted. Further review is not needed.

Comments:
This is a very interesting paper focusing on the issue of modeling the process of immobilizing protein enzymes on surfaces at a coarse-grained resolution, as well as the structural consequences of this process that determine enzyme activity. The described computational methodology itself is novel, albeit somewhat intuitive. However, the value of the paper is elevated by the following factors: (1) the thoroughness and systematic approach to the analysis, and (2) the multifaceted view of the problem, which goes beyond just conformational analysis and also includes indirect descriptions of enzymatic activity.

Response: We thank the referee for his/her favourable opinion about our paper and recommendation to publish, and for emphasizing our efforts to exploit the functional aspects in the coarse-grained modelling.

* The paper would benefit from a more detailed description of the experimental research section, especially its relevance in the context of simulation-based studies (which occupy the majority of the paper), as well as the significance of the results obtained. Currently, the information from the experimental part is almost absent in the main manuscript, and its existence is primarily indicated in the appropriate section in the Supplementary Information.

Response: In this work, we focused on previously reported experiments by Zeballos et al. (Ref. 40). For this reason, references to experimental data are intentionally brief. The core objective of this manuscript is the development and validation of the proposed simulation model; therefore, we direct readers to our previous publication, where the experimental methodology and comprehensive datasets are fully described.

In the present article, we limit the discussion to observable trends that emerge when comparing the simulated results with the experimentally reported data. Only a limited set of additional experiments was performed for this study, and these are appropriately described in the Supporting Information. We chose to include these experimental results in the Supporting Information instead of the main manuscript, as they are provided solely to support and contextualize the simulation outcomes rather than to constitute a primary experimental contribution.

* The weak point of the study, in my opinion, is the assumption of an ideally flat agarose surface. In reality, the agarose surface may be porous and contain various partially detached polysaccharide chains. The model adopted in the present work (the hexagonally arranged surface of the P4 Martini beads) is certainly a simplification. That said, I realize that creating a more realistic model may be challenging due to limited structural information. On the other hand, saccharide-specific parameters within Martini 3 exist, which makes it at least technically feasible. I suggest that the authors discuss the limitations of the model arising from this assumption.

Response: We thank the reviewer for this insightful comment and fully agree that representing the agarose support as an ideally flat surface is a simplification of the real, heterogeneous matrix. In this work, the flat P4-bead surface was deliberately chosen as a minimal and controlled model to isolate the effects of tethering geometry and orientation from confounding factors such as surface roughness or chain mobility.

Importantly, the supports employed experimentally are macroporous, with pore sizes typically exceeding 100 nm. Under these conditions, immobilized enzymes are anchored to the internal pore walls but remain in widely open environments, where physical confinement or curvature effects are minimal, except in rare cases where the enzyme is located at junctions of the crosslinked polymer network. Consequently, at the low protein surface densities considered here, a flat surface provides a reasonable approximation of the local environment experienced by the enzyme.

While more realistic agarose models are technically feasible within the Martini framework, their inclusion is hindered by the intrinsic heterogeneity of the matrix and limited structural information available, which would complicate a systematic comparison between immobilization strategies. Our primary objective was to investigate the physicochemical interactions between the enzyme and a hydrophilic, saccharide-based environment; given the composition of porous agarose, these sugar units dominate the nonspecific interactions with the enzyme once it is anchored via metal coordination bonds (as detailed in our response to Reviewer 1).

We have now explicitly updated the manuscript to discuss these limitations, clarifying that this representation serves as a reference model and that incorporating more complex surface architectures constitutes a natural direction for future studies. We say:

> *“While the flat surface utilized in this study provides a controlled environment to isolate the effects of tethering geometry, we acknowledge it represents a simplified*

model of the heterogeneous agarose matrix. This representation prioritizes the dominant physicochemical interactions between the enzyme and the hydrophilic saccharide units of the support, though it does not account for the inherent surface roughness or polymer chain mobility of the physical beads. Future iterations of this model could incorporate more complex surface architectures combined with saccharide specific

Martini parameters,[62] to further refine the description of the enzyme-support interface."

* This issue also has another aspect: would the procedure proposed by the authors be technically feasible for a different surface exhibiting different properties, such as charged saccharides, mineral materials, etc.? To what extent is the approximation of a flat, homogeneous surface adequate for this type of study?

Response: The modeling strategy proposed here is general and can, in principle, be extended to surfaces with different chemical properties, provided that appropriate coarse-grained interaction parameters are available. In this sense, the use of a flat, homogeneous surface should be regarded as a reference system rather than a universal representation of all immobilization supports. The flat-surface approximation allows us to decouple the effects of surface chemistry and tethering geometry from those of surface topology. This approach is particularly appropriate for addressing the central question of the present study —namely, how different immobilization sites and multivalent attachment strategies influence enzyme stability and function— independently of the specific nature of the support. We now clarify in the manuscript that while the quantitative results may depend on surface chemistry, the qualitative trends observed here, such as the localized rigidification induced by multivalent histidine clusters and the associated activity-stability trade-offs, are expected to be robust across different surface types.

* Is it possible that the lack of spontaneous enzyme adsorption on the surface (Fig. S1) is due to the absence of mobile anchors (polysaccharide chains) or porous surfaces, which would facilitate adsorption in realistic systems through their geometry?

Response: According to the design of our model, enzyme adsorption to the support surface occurs through interactions between the manually defined anchoring regions on the enzyme and the corresponding anchoring points on the support surface. In the absence of such anchoring elements (i.e., His-tags or His-clusters), spontaneous enzyme adsorption is rare. This behavior is consistent with the well-known inertness of agarose toward nonspecific protein binding, which is one of the reasons why agarose-based

materials are widely used in protein purification. Only in cases where enzymes are engineered to contain carbohydrate-binding motifs, such as lectins, can efficient immobilization on agarose-based materials be achieved (see, e.g., Peng et al, *ChemCatChem*, 2024; DOI:

10.1002/cctc.202400092).

In line with the reviewer's comment, we further note that the adsorption behavior in our simulations is sensitive to the physicochemical nature of the modeled surface. For more strongly interacting, highly hydrophilic surfaces (P1 bead type in Martini), spontaneous enzyme adsorption is clearly observed, as can be seen in Fig. R1 below. For the P4 surface, the protein-surface distance displays a more dynamic behavior, closer to that reported experimentally for agarose-based supports. These results indicate the lack of spontaneous adsorption reported in Fig. S1 reflects the intended surface chemistry of the model.

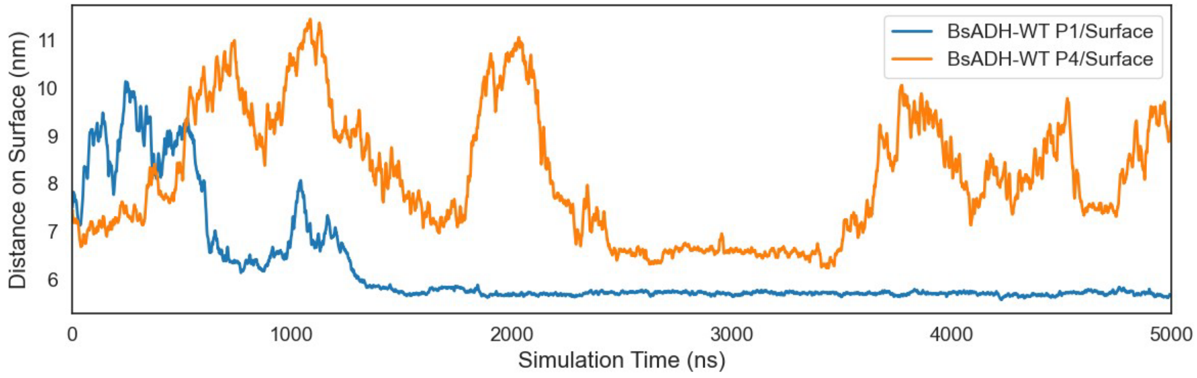

Figure R1: Time evolution of the protein–surface distance for BsADH adsorbed on a surface. The distance between the enzyme and the surface is shown as a function of simulation time for two representative adsorption surfaces (P1 and P4),

* The authors use the GoMartini approach to simulate the higher-order structure of proteins, which is a correct choice given the possible conformational changes induced by surface binding or temperature fluctuations. Do the authors believe that an alternative approach, such as using an elastic network model, would yield qualitatively consistent results with those described in the paper? Were such simulations carried out, for instance, in preliminary studies?

Response: We thank the reviewer for this insightful question. Elastic network models (ENMs) are widely used to describe equilibrium fluctuations around a native conformation. However, by construction, ENMs strongly constrain conformational flexibility through harmonic restraints and are therefore not well suited to capture the

large-amplitude, anharmonic motions induced by surface tethering or thermal perturbations, which are central to the present study. While many of the room temperature results would surely be recapitulated from an ENM, the partial unfolding events, subunit rearrangements, and loss of native contacts observed in our thermal stress simulations would be largely suppressed. As shown in our results (Fig. 4), these non-harmonic conformational responses are essential to discriminate between immobilization strategies with respect to enzyme stability and functional differences. For this reason, we selected the GōMartini approach, which preserves the native fold while still allowing unfolding and subunit reorganization.

Minor Points:

* Missing figure number in SI (Fig. ??).

Response: We thank the reviewer for pointing this out. The missing figure number in the Supporting Information has now been added.

* Some details regarding the pulling protocol may be worth mentioning: To which beads was the pulling force applied? What were the pulling rate and force constants in the pulling potential?

Response: We thank the reviewer for this suggestion. The immobilization protocol consisted of two stages. First, a steered pulling step along the surface normal (z-axis) was used to bring the enzyme close to the surface, using a pulling rate of 0.001 nm ps^{-1} and a harmonic force constant of 1000 kJ $mol^{-1}nm^{-2}$. Subsequently, the pulling was turned off and stable immobilization was modeled using static harmonic umbrella restraints applied to the histidine beads (terminal His-tags or engineered clusters) relative to a fixed agarose-like surface represented by P4-type beads. The restraints were applied at a target distance of 0.8 nm with the pulling rate set to zero. We have now clarified the details of the pulling protocol in the Supporting Information. We say:

> *"The immobilization protocol consisted of two sequential stages. In an initial deposition step, a steered pulling protocol was applied along the surface normal (Z-axis) to promote a rapid and controlled approach of the enzyme toward the surface, using a pulling rate of 0.001 nm. ps^{-1} and force constant of 1000 kJ .mol^{-1}. nm^{-2} .*

Once the enzyme reached the vicinity of the support, the pulling was switched off and tethering was modeled using static harmonic umbrella restraints. These restraints were

applied to the histidine beads involved in immobilization (either terminal His-tags or engineered histidine clusters), while the surface was represented by fixed P4-type beads mimicking an agarose-like support. A force constant 1000 kJ .mol^{-1}. nm^{-2} was used to restrain the histidine beads at a target distance of 0.8~nm from the surface, with the pulling rate set to zero."

Reviewer: 3

Recommendation: This paper is publishable subject to minor revisions noted. Further review is not needed.

Comments:
The work presented by Jimenez-Garcia et al. employs the widely used coarse-grained Martini 3 model to study conformational changes in protein systems. The study is novel and valuable, with potential for broad application across bioengineering and related fields, including those with more industrially oriented objectives. The authors have conducted and selected the methods that best approximate the problem under study. Moreover, structural and kinetic differences determined by MD simulations, among the systems analyzed, are clearly evidenced. Some comments that could help improve the manuscript are provided below.

Response: We thank the referee for his/her positive evaluation of our manuscript, the recommendation to publish and the valuable suggestions provided.

Main comments:

1) In Figure 1A, the structure could be better represented in cartoon format, with secondary structure elements labeled.

Response: We thank the referee for their useful suggestion. We have accordingly modified Fig. 1A and included the cartoon representation.

2) In Figure 1B, the chōon (ō) symbol should be used for GōMartini and other instances.

Response: We thank the referee for his/her attention to detail. We have now modified the figure and included the right symbol in the figure.

3) Could the authors specify how the contact map was determined in the study. Typically, the contact map (OV+rCSU) in GōMartini simulations is computed from an experimentally derived structure and then used for CG simulations.

Response: We thank the reviewer for this comment. The native contact map was computed from the experimentally determined crystal structure of BsADH (PDB ID: 1RJW) using the OV+rCSU scheme and then kept fixed for all GōMartini coarse-grained simulations. We have now clarified this explicitly in the main text. We say:

"In this work, native contacts were identified using the OV+rCSU scheme (see Fig. 1C-D), which combines geometric and chemical criteria for physical realism.[53] "

4) What are the limitations of considering the Martini P4-type as agarose-like? Additionally, later in paragraph 8 it is mentioned that anchoring without restraints does not show a stable interaction (protein/support). Indeed, the strategy used is interesting for modeling this type of system; however, it is important to clearly state the limitations of the model in terms of protein–surface interactions.

Response: We thank the reviewer for raising this important point. The Martini P4-type surface employed in this work is intended as a coarse-grained representation of an agarose-like support, capturing its highly hydrophilic and weakly interacting character rather than its full molecular complexity. As such, the model does not explicitly account for features such as mobile polysaccharide chains, surface porosity, or heterogeneous chemical functionalities, which may contribute to adsorption in real agarose-based materials.

Importantly, the absence of stable protein-surface interactions in the absence of defined anchoring elements is therefore an intrinsic and intended feature of the model, consistent with the experimentally well-known inertness of agarose toward nonspecific protein binding. This point has been addressed in detail in our response to the related comment from Reviewer 2 on spontaneous adsorption (see Fig. S1 and Fig. R1 above). In particular, we show that the adsorption behavior is highly sensitive to the physicochemical nature of the modeled surface: for more strongly interacting, highly hydrophilic surfaces (P1), spontaneous enzyme adsorption is clearly observed, whereas for the P4 surface the protein–surface distance displays a more dynamic behavior, closer to that reported experimentally for agarose-based supports.

These results highlight both the limitations and the strengths of the approach. While the P4 surface should not be interpreted as a chemically detailed agarose model, the

framework is general and flexible, enabling the systematic exploration of different surface chemistries and interaction strengths, as well as structurally patterned (non-flat) supports, within the resolution afforded by the coarse-grained representation.

5) In Figure 2B, since a frame at t = 50 ns is shown, it would be convenient to indicate in panel A the moment at which this transition occurs.

Response: We thank the reviewer for this helpful suggestion. We have now indicated in panel A the time corresponding to the structural snapshot shown at t = 50 ns in panel B by adding a vertical dashed line.

6) The GōMartini approach can have limitations regarding conformational sampling in multi-chain systems. In this case, the high stability observed could be due to an artifact resulting from a high number of contacts in the contact map. This could be discussed to highlight the limitations of the model.

Response: We thank the reviewer for highlighting this important point. We agree that the native-centric nature of the GōMartini model may limit conformational sampling in multi-chain systems. We have now explicitly discussed this limitation in the Conclusions section and clarified that our analysis focuses on comparative trends between variants rather than absolute stability values. We also highlight the availability of alternative native-centric Martini-based approaches and refined contact-map definitions that may further improve sampling in future studies. We say:

> *"Nevertheless, the GōMartini model is inherently native-centric and may restrict conformational sampling in multi-chain proteins, leading to a possible overestimation of absolute stability arising from the high number of stabilizing in the contact map. For this reason, the stability trends reported here are intended to be interpreted comparatively across variants rather than as absolute measures. Future developments in native-centric Martini-based models, including refined contact-map definitions [63,71], may further improve the description of thermal unfolding processes."*

7) It would be easier for the reader to identify the A and D subunits in Figures 3C–E. Moreover, are these letters (A–D) corresponding to domain names or only to chain IDs?

Response: We thank the reviewer for this comment. Subunits A–D refer to the individual BsADH chains as defined by the PDB chain IDs, and not to structural domains. Since the

chains are already consistently color-coded in Figures 3C–E, we have added a brief clarification in the figure caption to make this explicit.

8) Figure 3 could benefit from a SASA analysis of the whole system, comparing the His-tagged construct and the variants.

Response: We thank the reviewer for this valuable suggestion. Following this recommendation, we performed a SASA analysis for the full BsADH tetramer, resolving the contribution of each individual chain for the His-tagged construct and the H3 and H4 variants. The analysis reveals a clear and consistent trend observed in our main manuscript: in the H3 and H4 systems, chains A and D —those directly involved in surface attachment— exhibit systematically lower SASA values, reflecting their reduced solvent exposure upon immobilization (see Fig. R2 below). In contrast, the remaining chains are more solvent-exposed and display higher SASA values. For the His-tagged construct, all four chains show comparable SASA profiles, consistent with a more distant and less asymmetric interaction with the surface.

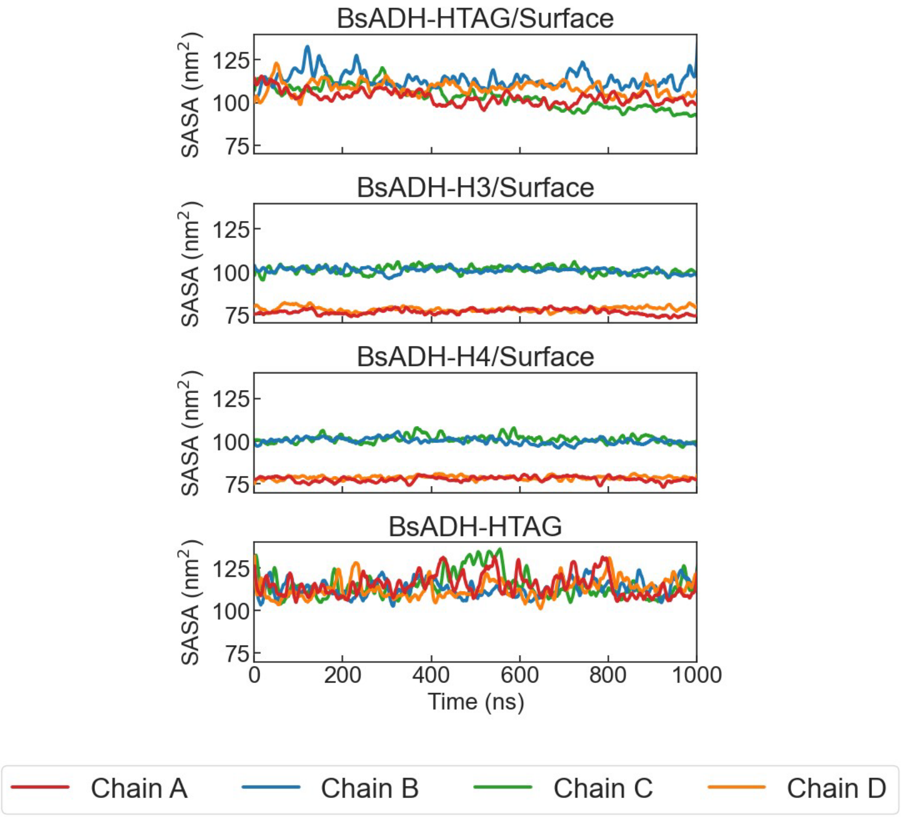

Figure R2: SASA analysis of the full BsADH tetramer for all immobilized variants and the free His-tagged system.

9) On page 4, in the third paragraph, the respective EtOH concentration could be indicated.

Response: We thank the reviewer for this suggestion. We have now explicitly indicated the ethanol concentration used to compute the association rate constants. This information has been added to the third paragraph on page 4.

"From the number of identified binding events, the ethanol association rate constant

(k_{on}) was estimated for each simulation run as $k_{on} = n_{bind} / (t_{sim} \cdot [\text{EtOH}])$, where n_{bind} is the number of binding events, t_{sim} is the simulation time, and [EtOH] is the ethanol concentration in the simulation box. This corresponds to an ethanol concentration of 6.34×10^{-3} M for the BsADH-HTAG system and 9.56×10^{-3} M for the BsADH-H3 and BsADH-H4 systems."

10) The authors mention that the modeling of EtOH binding and NADH release is well correlated with experimental data. Figure 5B could include experimental k_on values as a reference.

Response: We thank the reviewer for this suggestion. Experimental k_on values for ethanol binding to BsADH are not available. The experimental data reported by Zeballos et al.

(Figure S4 in Zeballos et al. *ACS Appl. Mater. Interfaces* 2024, DOI:

10.1021/acsami.3c15993) correspond to Michaelis–Menten parameters (k_{cat}, K_M, and k_{cat}/K_M), which reflect the overall catalytic efficiency and encompass multiple microscopic steps, including substrate binding, chemical conversion, and product release.

It should be emphasized that the Michaelis–Menten parameters were determined with respect to NAD^+; therefore, the ethanol k_{on} corresponds to a microscopic rate constant that cannot be derived from the apparent Michaelis–Menten kinetics obtained experimentally. Accessing such microkinetic parameters would require pre–steady-state measurements, such as stopped-flow experiments, which are technically extremely challenging when dealing with immobilized enzymes on microbeads.

These quantities cannot be directly compared to the microscopic association rates extracted from our simulations. Instead, our computational analysis separates two key steps that are expected to be most sensitive to immobilization: substrate entry and NADH

release. The observed trends in both quantities are consistent with the experimentally reported differences in apparent k_{cat}/K_M values, particularly the enhanced catalytic efficiency of the H3 variant and the reduced activity upon immobilization. For this reason, experimental k_{on} values could not be included in Figure 5B. We have clarified this point in the manuscript, explicitly indicating that the agreement with experiments refers to a qualitative correspondence between simulated substrate association and experimentally measured catalytic performance.

11) The aforementioned results are interesting. I would therefore recommend describing geometric parameters of the catalytic binding site, such as SASA or volume as a function of time.

Response: We thank the reviewer for this insightful suggestion. We have performed a geometric analysis of the catalytic binding site by monitoring the solvent-accessible surface area (SASA) of residues forming the cofactor-binding region as a function of time for all immobilized variants and for the free His-tagged enzyme.

This analysis reveals a clear distinction between immobilization strategies. In the H3 and H4 systems, the catalytic site of chains directly involved in surface attachment exhibits reduced SASA values, reflecting partial shielding of the cofactor-binding region due to oriented immobilization. In contrast, the remaining chains remain more solvent-exposed. For the His-tagged construct, all chains display comparable SASA profiles, consistent with a more symmetric and weakly constrained interaction with the surface. This geometric analysis supports and complements the dynamical and functional trends discussed in the main text.

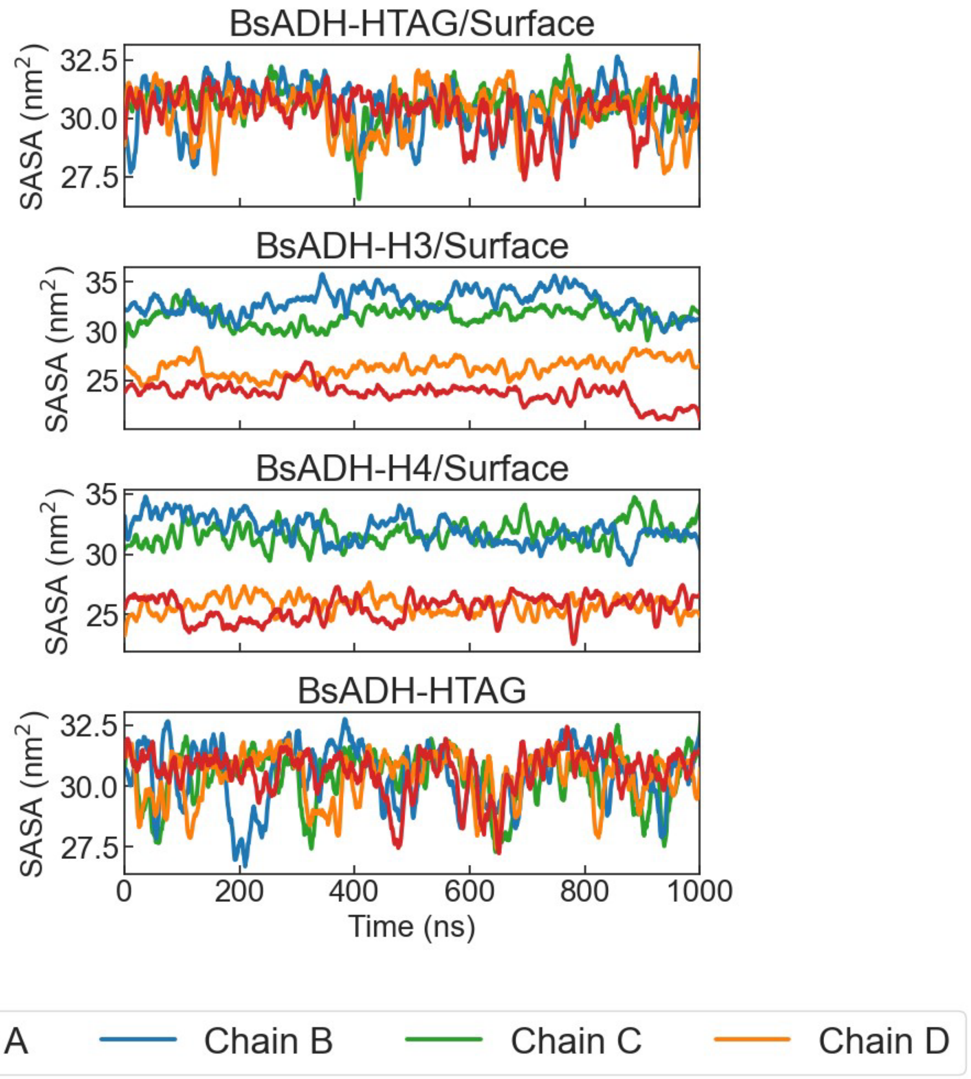

Figure R3. SASA analysis of the cofactor domain of BsADH tetramer for all immobilized variants and the free His-tagged system.

jz-2025-03753z.R2

Name: Peer Review Information for "Mechanistic Determinants of Oriented Enzyme Immobilization from Martini Simulations"

Second Round of Reviewer Comments

Reviewer: 2

Comments to the Author

All of my comments from the previous round of review have been taken into account, either in the responses to the reviews or in the content of the revised version of the paper. I have no further comments.

Reviewer: 1

Comments to the Author

I agree with the authors that several support-enzyme interactions are simultaneously occurring, there are not "main" group that can interact with the enzyme, mixed groups can promote interactions by all the components. The problem of this paper is that suggest that something very complex may be so simplified. If at least they evaluate using an inert support as starting point the effect of the growing concentrations of the groups, and the validate the model, this could be a more accurate model and the conclusions will be more supported.

As it is, even if I find the paper no conclusive enough, they should clearly reflect in the paper the simplifications, the possible interferences, and if using supports with growing concentrations of groups could help to advance or no. That is, this model can have some interest but give not a final answer to the effects of multieple enzyme-support interactions.

Author's Response to Peer Review Comments:

Reviewer: 1

Recommendation: This paper is publishable subject to minor revisions noted. Further review is not needed.

We thank the referee for judging that the reviewed article now requires only minor changes.

Comments:

I agree with the authors that several support-enzyme interactions are simultaneously occurring, there are not “main” group that can interact with the enzyme, mixed groups can promote interactions by all the components. The problem of this paper is that suggest that something very complex may be so simplified. If at least they evaluate using an inert support as starting point the effect of the growing concentrations of the groups, and the validate the model, this could be a more accurate model and the conclusions will be more supported. As it is, even if I find the paper no conclusive enough, they should clearly reflect in the paper the simplifications, the possible interferences, and if using supports with growing concentrations of groups could help to advance or no. That is, this model can have some interest but give not a final answer to the effects of multiple enzyme-support interactions.

We agree that our model aims to recapitulate a complex process such as enzyme immobilization. For this reason, several assumptions were necessary to construct the simulation model, and these assumptions are now clearly discussed in the revised manuscript. Among them, we already emphasized that the model represents flat surfaces (2D), whereas most agarose-based supports are three-dimensional porous materials. A second major assumption is that, once the enzyme is tethered to the support surface through His-mediated coordination bonds with metal chelates, nonspecific and weak interactions are dominated by agarose rather than by the few remaining cobalt chelates present at the surface after immobilization (see our response to the first round of revisions).

Importantly, we provide two major arguments that support this assumption. First, a P4 surface lacking anchoring points does not interact with the protein (as shown in Figure S1 of the Supporting Information), which is consistent with experimental data showing that neither His-tag nor His-cluster variants bind to macroporous agarose beads functionalized with cobalt chelates (see Figure R2.1 below).

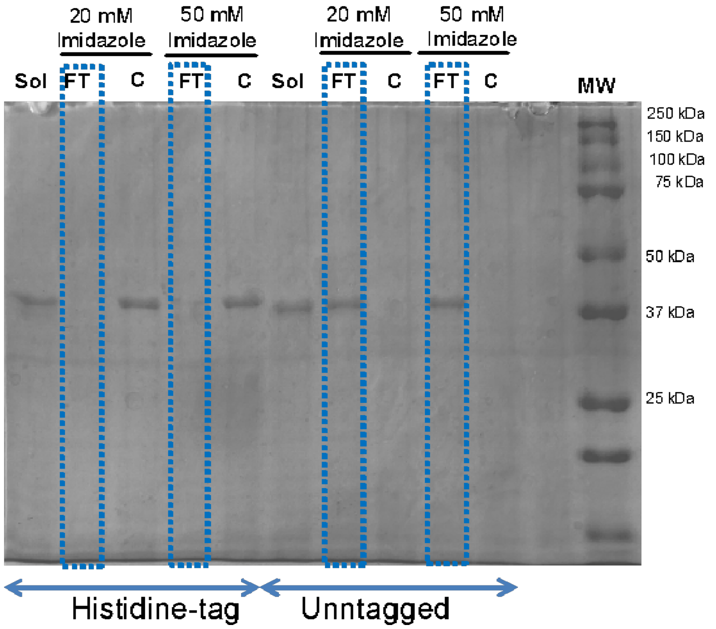

Figure R2.1 *(Adapted from ACS Appl. Mater. Interfaces 2024, 16, 1, 833–846). Immobilization of His-BsADH variant with (right) and without (left) His-tag immobilized on AG-Co2+ followed by SDS-PAGE. (Sol): Soluble variant offered to the carrier. (FT): Supernatant upon the immobilization of the variant in the presence of 20 mM and 50 mM imidazole; (C) AG-Co2+ carrier with the variant immobilized in the presence of 20 mM and 50 mM imidazole. The FT of all immobilization experiments is framed in blue.*

Second, simulating a surface in which the density of metal chelates exceeds that of agarose would not be realistic. Therefore, such a scenario was not considered in our model. To make this latter assumption more explicit, we have included the following sentence in the revised manuscript.

> In addition, our model assumes that cobalt chelates not involved in enzyme anchoring negligibly engage in nonspecific interactions with other residues exposed on the enzyme surface. This simplification was implemented because agarobiose repeating units are present at densities three orders of magnitude higher than cobalt chelates, based on the composition of 6% agarose microporous beads used in this study (nonspecific interactions between $^{-}$the surface and the enzyme are governed by the cobalt-chelate density = 15 μmol g^{1}). Consequently, we assume that the dominant agarose-like surface, simulated as P4, rather than by metal chelates. This assumption is consistent with observations reported in seminal studies on His-tag purification where only His-tagged proteins bind surfaces functionalized with metal chelates.
